# Combined Radiochemotherapy: Metalloproteinases Revisited

**DOI:** 10.3389/fonc.2021.676583

**Published:** 2021-05-13

**Authors:** Verena Waller, Martin Pruschy

**Affiliations:** Laboratory for Applied Radiobiology, Department of Radiation Oncology, University Hospital Zurich, University of Zurich, Zurich, Switzerland

**Keywords:** ionizing radiation (IR), metalloproteinases, combined treatment modalities, tumor microenvironment, radiotherapy

## Abstract

Besides cytotoxic DNA damage irradiation of tumor cells triggers multiple intra- and intercellular signaling processes, that are part of a multilayered, treatment-induced stress response at the unicellular and tumor pathophysiological level. These processes are intertwined with intrinsic and acquired resistance mechanisms to the toxic effects of ionizing radiation and thereby co-determine the tumor response to radiotherapy. Proteolysis of structural elements and bioactive signaling moieties represents a major class of posttranslational modifications regulating intra- and intercellular communication. Plasma membrane-located and secreted metalloproteinases comprise a family of metal-, usually zinc-, dependent endopeptidases and sheddases with a broad variety of substrates including components of the extracellular matrix, cyto- and chemokines, growth and pro-angiogenic factors. Thereby, metalloproteinases play an important role in matrix remodeling and auto- and paracrine intercellular communication regulating tumor growth, angiogenesis, immune cell infiltration, tumor cell dissemination, and subsequently the response to cancer treatment. While metalloproteinases have long been identified as promising target structures for anti-cancer agents, previous pharmaceutical approaches mostly failed due to unwanted side effects related to the structural similarities among the multiple family members. Nevertheless, targeting of metalloproteinases still represents an interesting rationale alone and in combination with other treatment modalities. Here, we will give an overview on the role of metalloproteinases in the irradiated tumor microenvironment and discuss the therapeutic potential of using more specific metalloproteinase inhibitors in combination with radiotherapy.

## Introduction

History of medicine assigns the first oncologic treatment with ionizing radiation to Emil H. Grubbe exposing the mammary carcinoma of Mrs. Rose Lee to X-rays in January 1896. Thereby the first milestone for a radiation-based treatment strategy was defined, which is indispensable nowadays for cancer therapy (1). Starting from low energy treatments of superficial melanomas towards high energy X-ray beams for the treatment of deeply located tumors in the early 20th century, the therapeutic use of radiotherapy (RT) has rapidly improved (2–4). Today, up to 50% of all cancer patients receive radiotherapy either alone, or in combination with surgery or systematic therapies (5, 6). The main rationale of radiotherapy is to achieve local tumor control by delivering a high dose of ionizing radiation to the tumor, while sparing the surrounding tissue and keeping the adjacent organs functionally intact.

Advances in intensity-modulated radiation therapy (IMRT) and image-guided radiation therapy (IGRT) paved the way towards better treatment planning and enhanced therapeutic efficacy (7). Despite being a highly localized treatment regimen with the ability to diminish tumors on a microscopic level, radiotherapy alone still fails to achieve tumor control for multiple tumor entities with many patients suffering from high tumor recurrence rates. Although overlooked for a long time, early studies already showed that tumor cells could exhibit intrinsic or acquired resistance mechanisms to ionizing radiation (IR), which are either due to the mutational status of the tumor or due to cellular and tumor pathophysiological processes induced by irradiation itself (8, 9). Tumors do not only consist of one malignant cancer cell population, but of a variety of different cell types and their sub-populations, which constitute the tumor microenvironment (TME). Only the deeper understanding of the heterogeneous architecture of the TME and its tight interplay with the tumor cells will lead us to the identification of related resistance mechanisms and novel treatment targets for a combined treatment strategy of radiotherapy with pharmacological agents (10).

In addition to DNA damage, IR also affects intra- and intercellular processes that trigger a multilayered stress response and co-determine the tumor response to RT. In this context, various signal transduction pathways are hijacked by the tumor for its cellular protection and are even further upregulated in response to irradiation. Among others, the MAPK axis represents one of the main signaling pathways controlling the majority of hallmarks of cancer, such as proliferative signaling, angiogenesis, inflammation and cell death evasion (11–13). Hence, upregulated kinase activity along those cascades leads to a proliferative advantage and cell survival upon IR. The basal phosphorylation status of a substrate is tightly regulated by the dynamic interplay between phosphatases and kinases. This interplay can be disturbed by reactive oxygen species (ROS) induced by IR. ROS oxidize critical cysteine residues in the conserved catalytic centers of phosphatases, thus impairing their function and shifting the balance towards a more phosphorylated and active state of the substrate (14, 15). Besides this ligand-independent activation of intracellular signal transduction cascades, ionizing radiation induces the secretion of growth factors and cytokines, thereby mediating intercellular communication via auto- and paracrine signaling through a wide range of soluble signaling molecules. Consequently, irradiation-induced secretion of pro-survival factors into the tumor environment also co-determines radiation resistance, as reported for multiple tumor entities including non-small cell lung cancer (NSCLC) and breast cancer (16, 17). In this review we will discuss the interplay between ionizing radiation and Zn-metalloproteinases, which represent the major class of proteases responsible for the processing of these secreted factors.

### Biochemistry of Metalloproteinases

Metalloproteinases are metal-, usually zinc-, dependent endopeptidases (metzincins) that play versatile roles in intercellular signaling pathways and tissue remodeling. The human superfamily comprises three subfamilies: matrixins (MMPs), astacins and adamalysins (18). Based on functional and structural properties, adamalysins can be further subdivided into a disintegrin and metalloproteinase (ADAM) and ADAM with thrombospondin motif (ADAMTS) (18).

Structurally the metzincins superfamily was defined by Bode et al. based on two properties which appear to be almost identical among all the members (19, 20). They reported an extended Zn^2+^ binding motif HEXXHXXGXXH in the catalytic site for the ligation of three zinc ions as well as a conserved methionine containing segment downstream of the third Zn^2+^-binding histidine, that supports the formation of a β-turn and therefore participates in the structural integrity of the catalytic domain (the Met-turn) (19, 21).

**Figure 1:**
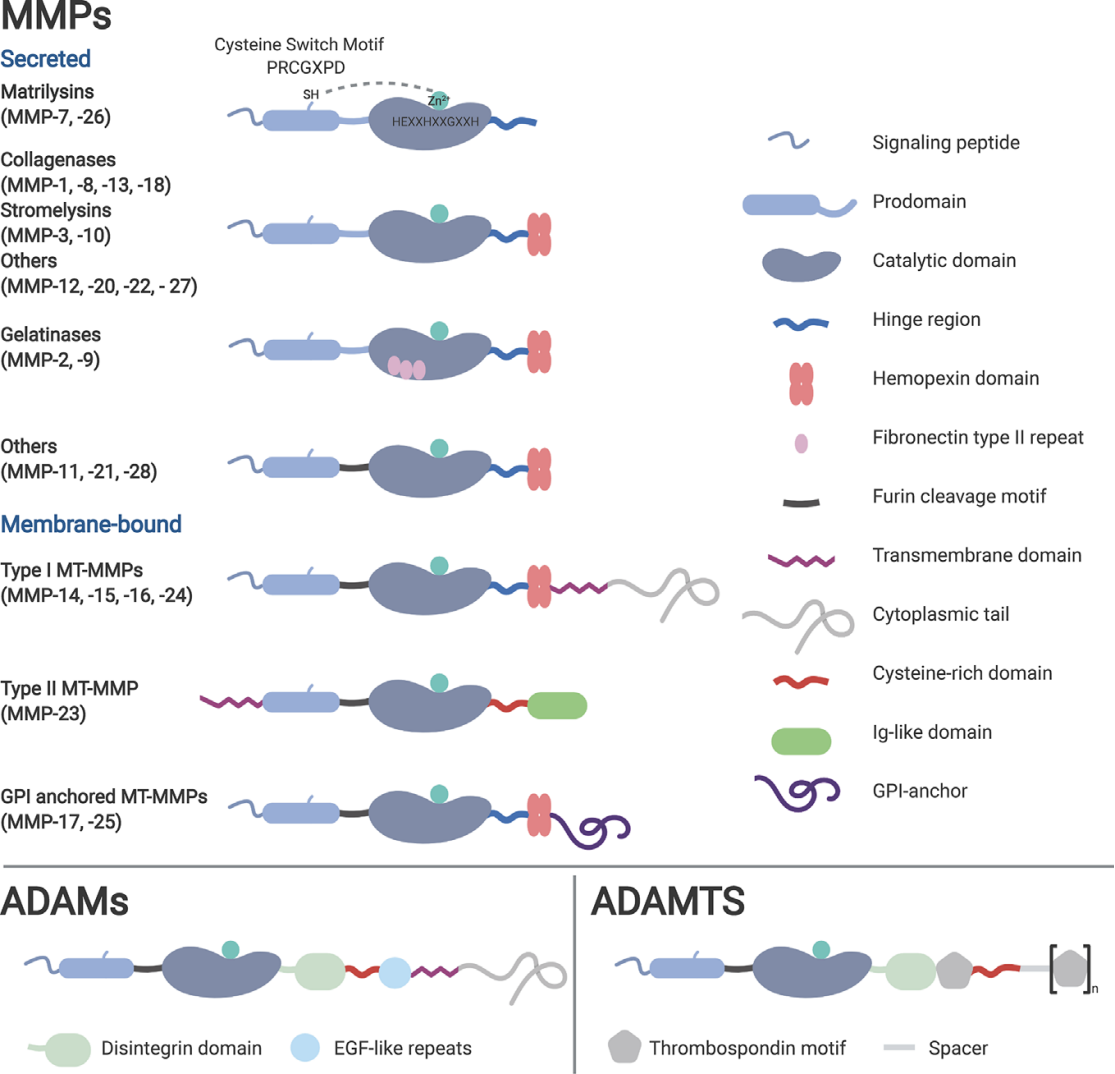
Classification of the metzincins based on their structure and function. Typical for metzincins is their signal peptide, the prodomain, the catalytic domain containing the zinc motif, followed by the linker (hinge) region. Membrane-associated metalloproteinases typically harbor a transmembrane and cytosolic domain, lacking in the secreted family members. Depending on their mode of activation, several metalloproteinases have a furin recognition sequence. Distinctive for many MMPs is the hemopexin (PEX) domain, which facilitates substrate specificity and TIMP interaction.

Besides those common features, the core structures among the subgroups are varying, depending on their function. While MMPs and ADAMTS are mainly involved in the remodeling of the extracellular matrix (ECM), most of the members of the ADAM family are actively associated with the process of proteolytic ‘shedding’ of membrane-bound proteins, hence the rapid modulation of key signals in the TME (22). Thus, MMPs and ADAMTS are mainly present as secreted enzymes within the ECM, while ADAMs typically remain membrane-associated (**Figure 1**).

In order to prevent dispensable protein degradation, most proteinases, hence also metalloproteinases, are synthesized as latent zymogens. The autoinhibitory propeptide harbors a seven amino acid long motif (PRCGXPD), with the thiol of the cysteine chelating with the active Zn^2+^ site of the catalytic domain of the protein, keeping it inactivated (23). Crucial for the activation of the latent proenzymes is the “cysteine switch”, a process describing the disruption of the thiol–zinc interaction (with or without cleavage of the propeptide) (24). This can be induced by the cleavage through other (metallo-) proteinases and allosteric disruption, or most commonly for the membrane-bound members of the metzincins, via proteolysis by the proprotein convertase furin (25). 

### Tissue Inhibitors of Metalloproteinases (TIMP)

Keeping the balance between an active and a latent state of metalloproteinases, four endogenous TIMPs (tissue inhibitors of metalloproteinases 1–4) are known to inhibit the active enzymes in mammalian tissue. Structurally, the small inhibitory molecules are highly conserved enabling them to inhibit all members of the metzincin family, but with different affinities and increased preference towards ADAMs and ADAMTS (26). TIMPs consist of two functional domains (stabilized via six disulfide bridges), that act independently from each other, pointing towards separate evolution (27). Most of the inhibitory capacity lies within the large amino terminus, whereas the role of the smaller carboxy tail is not well understood. Inhibition of the target takes place at the very end of the N-terminus (Cys1-Pro5) of mature TIMPs. This short peptide sequence forms five intermolecular hydrogen bonds within the active-site cleft, binding to the metalloproteinase in an almost substrate-like manner (28). The only known function of the carboxy-terminus is the formation of a non-covalent 1:1 complex with the hemopexin domain of proMMP-2/-9. Secreted as such, the complex remains stable and protected from degradation, while the amino terminus can still exhibit its inhibitory function on other MMPs (29).

## Matrix Metalloproteinases in The Irradiated Tumor Microenvironment

Already during the identification of the first matrix metalloproteinases a clear association with tumor progression was drawn, as various MMPs were found to be upregulated in human tumors (30). MMPs are mainly acting on the processing of extracellular matrix components, such as collagen, glycoproteins, and proteoglycans, and are highly distributed among different cell types and tissues. In terms of cancer progression, the increased abundance of active MMPs results in the disruption of the matrix barrier, enabling tumor cells to invade into the surrounding tissues and blood vessels. MMPs are therefore mainly discussed in the context of tumor dissemination. However, recent studies revisited the role of different MMPs, differentiating their mode of action into ECM processing versus non-matrix acting, leading to an increased focus on the MMP-regulated intercellular communication via the secretion of cyto- and chemokines, growth and pro-angiogenic factors (30, 31). The fine-tuned balance between MMP and TIMP activation controls this proteolytic shedding, which can be deregulated during cancer progression and in response to exogenous stress, such as ionizing irradiation. MMP maturation is a tightly regulated process in which RT can interfere on many levels. Besides direct cell killing, RT induces cellular and molecular changes within the TME that can activate MMPs. Overlooked for decades, immunologists have started to understand the immense ability of RT to induce a pro-inflammatory environment, susceptible for immune cell infiltration. No less important, the release of growth factors, chemo- and cytokines also has a direct impact on tumor stimulating MMP gene expression (32, 33). At the same time RT also increases furin gene expression, which results in increased furin-mediated posttranslational conversion of the proform and subsequent activation of many MMPs and all ADAMs/ADAMTS in response to irradiation (16, 25, 34). Furthermore, irradiation generates cellular reactive oxygen species, which also directly interact with biomolecules such as metalloproteinases. Metalloproteinase zymogens are regulated by the interaction of a cysteine amino acid residue in their prodomain and the Zn^2+^ site in the catalytic region. IR-induced oxidation of these critical cysteine sites leads to disruption of the inhibitory conformation and subsequent activation of the metalloproteinases (35, 36).

The diverse mechanisms by which irradiation influences the status of MMP activity in the irradiated TME renders this class of enzymes into an important family for the design of novel treatment strategies.

### Role of MMP-2 and MMP-9 for the Radiation Response

MMP-2 and -9 play crucial roles in ECM remodeling and cleavage of membrane substrates and have therefore been associated with several hallmarks of cancer such as angiogenesis, tumor invasion, and metastasis (37–41). Clinical studies identified MMP-9 as a potential prognostic biomarker for various tumor entities such as NSCLC (42), cervical cancer (43, 44), pancreatic cancer (45), and osteosarcoma (46). In many cases, elevated MMP-9 levels were associated with poor prognosis and decreased overall survival. In 2014, Yousef et al. also detected differential expression of MMP-9 in the different molecular subtypes of breast cancer. Importantly, MMP-9 overexpression was found to be an important endpoint for the more aggressive subtypes, triple-negative and HER2-positive breast cancers (41, 47).

Several studies reported irradiation-induced upregulation of MMP-9, which highly correlates with enhanced metastasis and cell invasiveness *in vitro* and *in vivo* and influences treatment outcome (48–51). Confirming increased MMP-9 levels upon sublethal irradiation of Lewis lung carcinoma, Chou et al. observed enhanced cell invasion *in vivo* that resulted in RT-induced acceleration of pulmonary metastases in their C57BL/6 mouse model. This effect could be inhibited by pre-treatment with zoledronic acid, a prototypical MMP-9 inhibitor. Interestingly, high-dose treatment (30 Gy) of the primary tumor decreased MMP-9 serum levels, improved tumor control and eliminated the amount of disseminating cells (48). In NSCLC cells (49) and hepatocellular carcinoma (50), irradiation enhanced MMP-9 expression via the PI3K/AKT/NF-κB and the PI3K/AKT/MAPK pathway, respectively, leading to enhanced tumor cell invasiveness. Investigating drivers of radioresistance, Ko et al. observed increased MMP-9 activity and elevated EMT protein levels in their RT-resistant breast cancer cell line (51). Thus, MMP-9 activity should be carefully probed as biomarker for putative irradiation-induced cell dissemination.

Interestingly, the relevance for potent MMP-9 inhibition as part of a combined treatment modality with RT has also been demonstrated on the systemic level. MMP-9 activity from bone marrow-derived CD11b-positive myelomonocytic cells was most relevant for the process of tumor vasculogenesis. Ahn et al. demonstrated that not endothelial progenitor cells but primarily tumor-site infiltrating CD11b+ myelomonocytic cells are involved in remodeling of the extracellular matrix in the irradiated tumor bed, in promoting vasculogenesis (instead of angiogenesis). They thereby represent a risk for local recurrences (52). Of note, genetic depletion of the respective metalloproteinase activity prevented tumor growth in these pre-irradiated areas. Eventually, these insights resulted in the promising development of anti-vasculogenesis strategies in combination with radiotherapy (53, 54).

In terms of clinical relevance, MMP-9 has been proposed as a predictive marker for the efficacy of radiotherapy in NSCLC. Serum of patients with intermediate and advanced stages of NSCLC were tested prior and after treatment [prescribed dose of planning target volume (50–66 Gy)] which was given in fractions of 1.8–2.0 Gy/day. Only in responders, the MMP-9 serum levels were significantly reduced at 1–5 weeks after treatment, whereas for patients with stable disease (SD) and progressive disease (PD) stage no changes in serum MMP-9 could be detected (55). An additional study on rectal cancer identified alterations in MMP-9 levels at different stages of treatment. Circulating MMP-9 levels were significantly reduced after induction neoadjuvant chemotherapy (NACT), gradually increased after sequential radiochemotherapy (RCT) and almost recovered to baseline 4 weeks after treatment. Notably, progression free survival (PFS) correlated with the initial drop of MMP-9 levels after NACT and RCT (56). One clinical study focused on the impact of radiotherapy-induced MMP-9 activation in the healthy tissue surrounding the targeted tumor. After neoadjuvant RCT of esophageal cancer patients MMP-9 levels increased in the proximal and even distal healthy esophageal tissue, which could be associated with post-operative complications such as anastomotic leakage, and could potentially be avoided by MMP-9 inhibition (57).

Due to their structural and functional similarities, it is not surprising that multiple studies report co-upregulation of MMP-9 and MMP-2 upon irradiation, leading to increased tumor cell invasiveness, metastasis, and angiogenesis. MMP-2, which belongs to the same gelatinase family as MMP-9, is also highly associated with various tumor entities such as prostate cancer (58), gastrointestinal carcinomas (59, 60), and cervical cancer (44, 61). Similar to MMP-9, IR also induces upregulation of MMP-2 resulting in enhanced tumor growth and cell invasiveness. Moreover, MMP-2 activity is required for the angiogenic switch during tumor development and has, together with MMP-9, been implicated in the regulation of expression and release of vascular endothelial growth factor (VEGF) (62–65). Combining RT with inhibition of MMP-2 activity impaired cancer cell invasion, reduced VEGF secretion and hindered radiation-induced capillary tube formation *in vivo*, expanding its role to an important regulator of angiogenesis (63, 64).

Bidirectional activation between MMP-2 and the pro-survival transcription factor FoxM1 influenced cell cycle progression and thereby impacted the treatment outcome of DNA damaging agents. Inhibition of MMP-2 abrogated IR-induced FoxM1 expression to overcome G2/M cell cycle arrest, thereby driving cells into apoptosis (66).

Genotyping of patients with advanced stages of NSCLC after RT revealed that carriers of selected functional MMP-2 polymorphism had significantly reduced PFS, proposing MMP-2 as prognostic marker (67). In glioma cells, RT-induced secretion of MMP-2/-9 enhanced tumor cell migration *in vitro* and dissemination *in vivo*. Conversely, TIMP-2 protein expression, which antagonizes MMP activation, was strongly reduced (68). In breast cancer and rectal cancer specimen, MMP-2/-9 activation was observed to be enhanced in the tumor site in comparison to the adjacent healthy tissue, and correlated with dissemination, cancer progression and treatment outcome (69–71). After radiotherapy, MMP-2/-9 levels increased drastically within the rectal adenocarcinoma indicating a role of MMP-2/-9 for radioresistance (71). Taken together, serum and even urine levels of circulating MMP-2/-9 can be used as good clinical markers for tumor incidence, cancer stages, and treatment prognosis or success (70, 72–74). At the same time, specific MMP-2/-9 inhibitors could be promising radiosensitizers in cancer therapy.

### Versatile Roles of Other MMP Family Members

Even though MMP-2 and MMP-9 represent the most investigated MMPs in the context of radiotherapy, also other family members have been associated with the remodeling of the irradiated tumor microenvironment. Indeed, one of the first *in vivo* studies combining MMP inhibition with radiotherapy was conducted in 1992, with Sotomayor et al., detecting increased tumor growth control upon treatment with the collagenase (MMP-1) inhibitor minocycline in combination with RT (75). In addition to enhanced rates of cancer cell intravasation and dissemination, RT-induced MMP-activation (MMP-1/-2/-3/-9/-14) and subsequent degradation of the TME and the mucosal tissue adjacent to the irradiated tumor site, can induce strong normal tissue toxicities (76, 77). Elevated levels of secreted MMP-1/-2/-9 in the mucosa of rectal cancer patients after RT resulted in gut tissue toxicity increasing the risk for post-operative morbidity, wound infections as well as metastasis formation (78, 79). Interestingly, several studies also demonstrated an increase in MMP-7 gene expression after surgery and pre-operative high-dose RT in colorectal carcinoma cells but not in the adjacent mucosal tissue (80–82). Furthermore RT affected MMP-7 expression in a dose dependent way indicating that MMP-7 levels are very sensitive to different types of trauma, which can define treatment outcome and resistance (82). Hence, different MMPs are responding in a differential way to radiotherapy and combining radiotherapy with specific MMP inhibitors could not only decrease the risk of local tumor recurrence but could also protect the healthy mucosa.

In oral squamous cell carcinoma patients, the MMP-13 expression levels highly correlated with different clinicopathological parameters, such as staging and grading of the tumor. Additionally, patients harboring less MMP-13 transcripts showed a better treatment response to radiotherapy in comparison to patients overexpressing MMP-13 (83). Similar results were obtained in a glioma patient study indicating its potential use as a predictive biomarker for RT, while another study determined MMP-13 as prognostic marker for tumor aggressiveness and recurrence in head and neck cancer patients (84, 85).

Among the six known membrane anchored matrix metalloproteinases, membrane type I matrix metalloproteinase MT1-MMP (MMP-14) is highly associated with cancer progression, angiogenesis, and immune response (86–89). Besides its original role as collagenase and MMP-2 activator, proteomics analysis of human melanoma cells revealed a broad influence of MT1-MMP on the tumor microenvironment, based on the shedding of a variety of adhesion molecules, receptor and transporter proteins (90). MT1-MMP accumulates on the migratory front of cells and facilitates the degradation of collagen, fibronectin and CD44. Disruption of the ECM barrier enables cell motility, and therefore MT1-MMP was considered as important protease for (tumor) cell migration and invasion (87, 91). Thus, MT1-MMP is an interesting target in combination with RT to mitigate cell migration and metastasis formation. In breast cancer models inhibition of MT1-MMP synergized with ionizing radiation and reduced cell migration (92–94). Investigating the invasiveness of triple-negative (TN) breast cancer cells after RT, MT1-MMP downregulation reduced the number of circulating tumor cells and lung metastases (93). Besides its pro-migratory effect, MT1-MMP has also been identified as an activator of the immune-suppressive cytokine transforming growth factor (TGF) β (95). Consistent with the decrease of TGF-β secretion, blockade of MT1-MMP with the antibody DX2400 polarized tumor-associated M2-like macrophages towards the anti-tumor M1-like population, contributing to tumor growth delay and reduced necrosis (94). In addition, MT1-MMP inhibition improved vessel perfusion and oxygenation of the tumor. Overcoming tumor hypoxia is one of the main challenges in the field of RT as hypoxic cells are radioresistant and negatively influence treatment outcome (96). Thus, MT1-MMP represents an interesting target in particular for the combined treatment of hypoxic tumors.

Moreover, an interesting study on intracellular signaling extended the effect of MT1-MMP beyond the tumor microenvironment and proposed its involvement in the DNA damage response along the MT1-MMP-integrinβ1 pathway (97). Inhibition of MT1-MMP reduced integrinβ1 signaling and sensitized TN breast cancer cells to radio-and chemotherapy by collapsing the replication machinery. Thus, combining RT with MT1-MMP inhibition could not only prevent cell dissemination but also enhance direct DNA damage.

Our own studies on increased MMP activities and invasiveness of irradiated tumor cells exemplify the complex network and regulation of metalloproteinase activities in response to stress. The IR-enhanced invasive capacity of fibrosarcoma and glioblastoma cells could mechanistically be linked to increased MMP activities, though irradiation only partially increased expression of MMP-2/-9/-14. On the other hand, irradiation specifically induced the secretion of TIMP-1/-2. Depending on the ratio of TIMPs and MMPs, TIMPs can not only inhibit but also activate MMPs, with TIMP-2 being relevant for processing of pro-MMP-2 (98). Interestingly, downregulation of TIMP-1/-2 not only reduced respective MMP-activities but also specifically blocked IR-induced invasiveness of these irradiated tumor cells. Cell invasion induced by low radiation doses (1.5–2.0 Gy) is of particular importance in the context of fractionated radiation schedules and sub-lethal irradiation of peripheral tumor cells of the radiotherapy treatment volume. Thus, a combined treatment modality reducing IR-upregulated MMP might reduce the potential risk for IR-induced (glioma) cell migration and dissemination. 

## Adam-Induced Secretome in The Irradiated Tumor Microenvironment

In the past decades, the importance of the ADAM family members has shifted from embryonic development to versatile roles in disease including neurodegeneration, inflammation, and cancer in particular. ADAMs are recognized as important players in the ErbB1 (EGFR) signaling axis as ADAMs shed a large variety of (mitogenic) growth factors, growth factor receptors and cyto- and chemokines. The ErbB1 pathway is associated with cancer growth and progression and represents an attractive target for cancer therapy. However, targeting the pathway directly with tyrosine kinase inhibitors such as gefitinib has been challenging due to acquired pro-resistance mutations (99). The combination of RT with the ErbB1-directed monoclonal antibody cetuximab improved locoregional control and survival of patients suffering of advanced squamous-cell carcinoma of the head and neck, whereas the trimodal treatment with chemoradiotherapy and cetuximab showed no additive beneficial effect in stage III NSCLC patients (100, 101). Therefore, it could be of interest to inhibit not only ErbB1- but multiple ErbB (ErbB1–4), and other related receptor tyrosine kinases and signal transduction cascades, via inhibition of upstream sheddases such as ADAMs. ADAMs are upregulated in many cancer entities and have been associated with promotion of cell growth, survival, migration, and invasion (102). Similar to MMPs, ADAMs are activated in multiple ways, including gene expression, translocation to the cell membrane, posttranslational modifications on the cytoplasmic tail, zymogen activation via furin or their interplay with TIMPs (102, 103).

Among all ADAMs, ADAM10 and ADAM17 share the most structural and functional properties, being best known for their role in Notch signaling and the clinicopathology of Alzheimer’s disease. ADAM17 represents the most intensively studied member of the ADAMs family and gained attention especially in the context of inflammatory disease due to its processing of TNF-α. Thus, ADAM17 is also known as TNF-alpha converting enzyme (TACE). As part of our own TME-oriented research we investigated how RT-induced secretion of para- and autocrine stress-response factors modulates cellular radiosensitivity, drives acquired rescue mechanisms and determines the overall radiation sensitivity of a tumor. We performed exhaustive large-scale secretome analysis using antibody arrays for a wide range of secretory factors (16). Secretion kinetics of selected factors were determined across different established tumor cells and in murine blood serum, derived from irradiated tumor xenograft-carrying mice. RT-induced expression and tumor cell secretion included top hits, such as amphiregulin, TGF-α and ALCAM. All these factors were secreted in a similar RT-induced time- and dose-dependent manner from several NSCLC cell lines (and other tumor entities), indicative of a common upstream mechanism without changes at the transcriptional level, pointing towards ADAM17. Interestingly, irradiation induced a dose-dependent increase in cleavage of the proform of ADAM17 by furin, which resulted in enhanced ADAM17 activity and correlated with subsequent substrate shedding. Pharmacological inhibition of ADAM17 with the small molecular inhibitor TMI-005 or siRNA-based targeting of ADAM17 suppressed RT-induced shedding of these factors, downregulated ErbB1-signaling in target cells and enhanced RT-induced cytotoxicity *in vitro* and *in vivo* (tumor xenograft model) even in tumors resistant to ErbB-targeting cancer therapeutics. *Ex vivo* substrate analysis of murine blood serum derived from irradiated tumor xenograft-carrying mice correlated with our *in vitro* results. Not surprisingly the supra-additive response to the combined treatment modality of RT and inhibition of ADAM17 on the *in vivo* level point towards multiple mechanisms of action, including tumor cell- and TME-oriented ionizing radiation-sensitive processes.

Cancer stem cell are often characterized by increased radiation resistance (104). Investigating the radioresistant and migratory phenotype of CD133+ liver cancer stem cell (CSC) Hong et al. observed next to increased MMP-9 and -2 expression also IR-enhanced ADAM17 activity in the CD133+ enriched cell population of hepatocellular carcinoma (HCC) (105). Of note inhibition of ADAM17 sensitized these CSCs to IR and disrupted their IR-induced metastatic potential. Overall, ADAM17 is gaining recognition in the field of combined treatment modalities with RT, in particular for aggressive tumor entities with high recurrence rates.

Even though ADAM10 and respective inhibitors are highly discussed as novel targets for cancer treatment, ADAM10 has not been very much investigated in combination with radiotherapy. As depicted by Sharma et al., while ADAM17 activity increased in an IR-dose-dependent manner, irradiation of NSCLC cells did not upregulate ADAM10 activity in these cells (16). However, IR-induced upregulation of these ADAM-isoforms might be tumor entity dependent. In a very recent report, Mueller et al. demonstrated IR-increased ADAM10 expression in pancreatic tumor cells, which correlated with RT-induced fibrosis, tumor cell migration, and invasion. Targeting of ADAM10 sensitized orthotopic tumors to IR and prolonged mouse survival (106). Furthermore, a putative risk for cardiovascular damage exists as exposure of endothelial cells to irradiation increased the levels of active ADAM10 in those cells (107, 108). Subsequently ADAM10-mediated degradation of the endothelial specific adherens junction VE-Cadherin resulted in increased vascular permeability. Weakening the endothelial barriers facilitates transendothelial tumor cell migration and dissemination but also ischemic disease after RT. Thus, it is important to appreciate the vascular system as an organ of risk when irradiating solid tumors.

Most studies investigating the response of ADAMs to IR in cancer and adjacent endothelial tissues observed an upregulation of the metalloproteinases on the expression, total protein and/or activity level. However, studying radiation-induced renal dysfunction and tissue toxicity in healthy renal epithelial cells revealed the opposite effect. IR induced a downregulation of ADAM9/10/17 *in vitro* (mIMCD-3 cell line) as well as in kidney tissue derived from BALB/c mice. This phenotype directly correlated with decreased levels of the soluble anti-aging suppressor Klotho, a substrate of ADAM9/10/17. The clinical consequences are premature cellular senescence, nephropathy and even kidney failure as severe side effects after RT (109).

The reduction of the oxygen partial pressure below a critical physiological level represents a major radioresistance mechanism in tumors, due to the altered physico-chemical conditions but also due to biological adaptations. Tumor hypoxia renders tumor cells up to threefold more radioresistant than their normoxic counterparts. The hypoxia-inducible factor (HIF)-1α is stabilized under hypoxic conditions, accumulates and transactivates a large variety genes involved in the adaptive response of the tumor cells to hypoxia, including genes involved in metabolism, angiogenesis, cell proliferation, and also different metalloproteinases (110–112). Direct (through binding to the respective promotor region) and indirect mechanistic links were identified between HIF-1α accumulation and increased gene expression of MMP-1/-9/-13/-14 as well as ADAM10/17 (111–117) and correlated in most cancer cell types with increased aggressiveness and invasiveness (111–113). To ensure energy sustainability, hypoxic cancer cells shift their metabolism towards the glycolysis pathway (118), which generates high amounts of acidic end products. One important part of the pH-regulatory machinery plays the tumor-associated zinc-metalloenzyme carbonic anhydrase IX (CAIX) (118). Induced by HIF-1α, high levels of membrane-bound CAIX have been associated with cancer cell invasiveness and therapeutic resistance. Interestingly, hypoxia-stabilized HIF-1α also promotes increased ADAM17 expression (117), which recognizes CAIX on the cell surface as a substrate and releases the enzymatically active ectodomain of CAIX (119). However, the consequences of this specific altered extracellular proteome for the exact pro- and anti-tumorigenic responses have yet to be investigated (120).

Next to the release of immunosuppressive factors, a hypoxic tumor microenvironment also impairs anti-cancer immunity through HIF-1α-mediated upregulation of ADAM10. ADAM10 is required for shedding of MHC class I chain-related molecule A (MICA), which activates natural killer (NK) cell effector function and cell lysis. Decreased levels of MICA under hypoxic conditions subsequently lead to immune escape and tumor cell resistance to the cytolytic action of innate immune effectors (116). Due to their wide range of substrates, their importance for Notch signaling pathways and as attributes of almost every cell type of the immune system, ADAM10 and 17 have gained particular attention in recent years in immunology research (121). Furthermore, ADAM17 is considered the main protease to cleave the Fcγ receptor CD16A (FcγRIIIA) on NK cells, which is involved in antibody-dependent cell-mediated cytotoxicity (ADCC) (122, 123). Human NK cells exclusively recognize tumor-targeting therapeutic monoclonal antibodies via intact CD16A. Engagement with the target cell induces NK cell degranulation, followed by the release of cytolytic granules (124). Complementary to this, ADAM17 also cleaves CD62L (L-Selectin), an adhesion molecule that facilitates mobility and homing of lymphocytes, including NK cells (122). Furthermore, NKG2D ligands are also substrates of ADAM17, and as such ADAM17 plays a major role in the regulation of the innate immune system through direct cell killing (natural cytotoxity) (125, 126). Hence, inhibition of ADAM17 on tumor cells and NK cells could strongly enhance anti-tumor immunity alone and as part of combined treatment modalities with different targeting agents and immunogenic cell death inducers. Moreover, ADAM17-mediated shedding has also been investigated in CD8+ T-cells towards activation of proliferation but also as inducer of apoptosis (127, 128). CD62L shedding positively affected early clonal expansion of cytotoxic T-cells in virus-transfected mice suggesting ADAM17 as an important regulator of T-cell activation (127). Recent studies also identified the programmed death ligand 1 (PD-L1) as a novel substrate of ADAM17 (128, 129). Taken together, novel immunotherapeutic approaches should carefully consider the role of ADAMs as additional immune regulatory target and resistance mechanism to immune checkpoint inhibition, also when combined with radiotherapy.

The plethora of molecular interdependencies between tumor hypoxia, the immune system and radiotherapy is not within the scope of this review and is summarized elsewhere (130–133).

## Targeting Metalloproteinases for Cancer Treatment

In addition to the use of metalloproteinases as diagnostic markers for tumor prognosis and treatment prediction, many efforts towards the development of potent MMP/ADAM inhibitors were pursued - unfortunately only with minimal success, which is primarily due to the lack of high specificity. Nevertheless, we will summarize the major developments on the preclinical and clinical level and point towards combined treatment modalities with radiotherapy.

### Small Molecular Inhibitors

**Figure 2:**
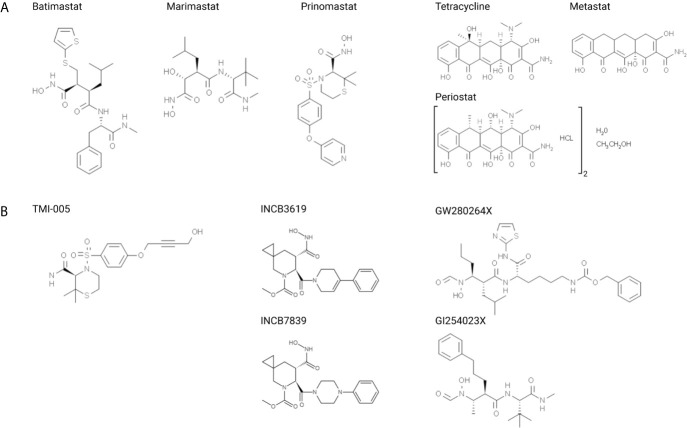
Structural formulas of small molecular inhibitors discussed in this review, divided into **(A)** MMP-directed and **(B)** ADAM-directed inhibitors.

The first generation of small molecular inhibitors comprised two functional groups: hydroxamic acid motifs that target the catalytic site of the MMPs by chelating the active zinc ion and a peptide derivate mimicking the collagen binding motif (**Figure 2**) (134). Binding to those peptidomimetics changes the conformation of the catalytic domain of metalloproteinases and disrupts the integrity of the enzyme. Batimastat (BB-94) was the first MMP-inhibitor to enter clinical trials (135). However, it could not be orally administered, thus, clinical testing was discontinued (136). The structurally related and orally bioavailable Marimastat (BB-2516) achieved promising results in early clinical trials (**Table 1**) (31). Nonetheless, in phase III clinical trials for different cancer entities Marimastat did not show added survival benefit and many treated patients suffered severe musculoskeletal side effects (31, 137). This high tissue toxicity is most probably due to the low selectivity of these broad-spectrum inhibitors towards different zinc-dependent proteases (137). Lessons from those early therapeutic efforts resulted in compounds targeting unique structural properties of MMPs. One structural characteristic that next generation of MMP inhibitors took advantage of was the variable S1’ pocket of metalloproteinases. This pocket lies in close proximity to the Zn^2+^ binding site in the catalytic domain and defines binding and substrate specificity (134). Based on amino acid variation on this primed enzyme site MMPs can be classified into “deep pocket” and “shallow pocket” enzymes (134, 138). The majority of MMPs harbor a leucine that forms their S1’pocket, resulting in an open conformation, whereas the small pocket for MMP-1/-7/-11 is partially or entirely occluded by larger amino acid residues (arginine, tyrosine, and glutamine, respectively) (134). The design of the nonpeptidic collagen-mimicking inhibitor Prinomastat (AG3340) was based on this rationale resulting in enhanced specificity for “deep pocket” MMP-2/-3/-9/-13. However, in phase III trials of advanced lung or prostate cancer Prinomastat did not improve clinical outcome when combined with chemotherapy, and further clinical studies were halted (31, 139).

**Table 1:** Summary of discussed metalloproteinase inhibitors in cancer-related clinical trials.

Name	Target	Tumor entity	Identifier
Marimastat (BB-2516)	Broad spectrum	SCLC	*NCT00003011*
		NSCLC	*NCT00002911*
		Breast cancer	*NCT00003010*
Prinomastat (AG3340)	MMP-2/-3/-9/-13	NSCLC	*NCT00004199*
		Brain and Central Nervous System Tumors *plus RT*	*NCT00004200*
		Prostate cancer	*NCT00003343*
Neovastat (AE-941)	MMP-2/-9/-12	NSCLC *plus RT*	*NCT00005838*
		Multiple Myeloma	*NCT00022282*
		Kidney cancer	*NCT00005995*
Metastat (Col-3, Incyclinide)	MMP-2, MMP-9	AIDS-Related Kaposi’s Sarcoma	*NCT00020683*
		Advanced Solid Malignancies	*NCT00003721*
		Refractory metastatic cancer	*NCT00001683*
		Brain and Central Nervous System Tumors	*NCT00004147*
INCB7839 (Aderbasib)	ADAM10, ADAM17	Gliomas	*NCT04295759*
		Diffuse Large B Cell Non-Hodgkin Lymphoma	*NCT02141451*
		HER2+ metastatic Breast Cancer	*NCT01254136*
			*NCT00864175*
		Solid Tumors	*NCT00820560*
Andecaliximab (GS-5745)	MMP-9	Gastric or Gastroesophageal Junction Adenocarcinoma	*NCT02864381*
			*NCT02545504*
			*NCT02862535*
		Advanced solid tumors	*NCT01803282*
		Glioblastoma	*NCT03631836*

TMI-005 (Apratastat), which shares structural similarities with Prinomastat, was originally designed for the treatment of rheumatoid arthritis due to its inhibitory potential of TNF-α release (140, 141). In contrast to previous clinical investigations with other small molecular inhibitors, TMI-005 showed very low tissue toxicity but the program with TMI-005 and other closely related derivatives was stopped due to lack of efficacy, related to constitutive activation of the TNF receptor on immunological cells, but not due to toxicity reasons (140). Based on its low toxicity profile and target relevance independent of TNF-α, TMI-005 and other new classes of ADAM17 inhibitors thus have a strong rationale for repurposing as drug in cancer therapy. After identifying ADAM17 as an important player for radiation resistance in NSCLC cells, our own studies demonstrated that pretreatment with TMI-005 sensitized NSCLC cell lines to RT and reduced secretion of ADAM17-specific substrates (16). Determining its efficacy in NSCLC cell-derived xenografts revealed supra-additive tumor control in combination with RT and defines its potential in cancer therapy.

Several other small molecular inhibitors have been designed to target members of the ADAM family with increased affinity towards ADAM10 and ADAM17. These two sheddases act upstream of multiple ErbB pathways, and interestingly their inhibition also synergized with therapeutics agents directly targeting the ErbB (1–3) pathways. Combining INCB3619, a dual inhibitor against ADAM10/17, with gefinitib or paclitaxel strongly downregulated proliferation of NSCLC cells, whereas other cell lines, which proliferate independently of ErbB-signaling, remained unaffected (142). Also in breast cancer models, selected sheddase inhibition mitigated the release of ErbB family ligands and enhanced the effect of ErbB-directed therapies *in vivo* (143, 144).The structurally related but pharmacokinetically improved inhibitor INCB7839 underwent clinical trials for the treatment of HER2 (ErbB2)-positive breast cancer patients with an interesting rationale to overcome trastuzumab-resistance. HER2 is a substrate of ADAM10 and the ADAM10 inhibitor INCB7839 reduced cleavage and release of the extracellular domain, thereby overcoming resistance to HER2-directed trastuzumab (145, 146). Indeed, administration of INCB7839 was well tolerated, decreased the plasma level of the extracellular domain of HER2. Future trials will show whether the promising combined treatment strategy will improve clinical outcome. HER2-mediated resistance mechanisms to other pharmacological therapies also exist in colorectal cancer cells and could also be related to upregulated ADAM10/17 (147, 148). As such treatment of colorectal cancer cells with the dual ADAM10/17 inhibitor GW280264X sensitized cells to chemotherapy (5-FU) (148).

Investigating the involvement of ADAM10/17 in the immunogenicity of glioblastoma-initiating cells, Wolpert et al. determined their role in regulating the NKG2D receptor-ligand system (among others MICA, MICB, ULBP2) (149). Inhibition of ADAM10/17 with GW280264X and the more specific ADAM10-directed compound GI254023X, increased cell surface abundance of ULBP2, which directly resulted in an increased immune response and susceptibility for NK cell mediated lysis. Studying the effect of ADAM10/17 inhibition on irradiation-induced cell permeability of endothelial cells, GI254023X revealed the strong involvement of ADAM10 in VE-cadherin regulation and transendothelial migration (108). Overall, these mechanisms point towards the versatile function that ADAM10/17 exert in the tumor microenvironment.

Shortly after identifying shark cartilage as the first tissue with anti-angiogenic (anti-MMP-2/-9/-13) properties, the functionally active, naturally occurring compounds were extracted and developed as Neovastat (AE-941) (150). *In vivo*, treatment with Neovastat alone showed inhibited neovascularization and metastasis formation in a Lewis lung carcinoma model. Combination with cisplatin increased the therapeutic index showing strong anti-metastatic effects while protecting against cisplatin-induced myelosuppression (151). Being introduced to phase I/II trials, Neovastat was well tolerated and demonstrated increased median survival in patients with solid tumors, including renal, prostate and lung carcinoma (150). However, in patients with unresecetable stage III NSCLC, the treatment with Neovastat to did not improve efficacy of chemoradiotherapy and has not been recommended for further treatment of lung cancer (152).

Apart from their conventional role as antibiotics, tetracyclines are effective inhibitors of metalloproteinases in the treatment of malignant disease. Early studies suggested non-antimicrobial functions of synthetic tetracyclines as inhibitors of collagenase and gelatinase activity in periodontitis and anti-proliferative and anti-migratory effects migration in cancer cells (153–156). This new, promising function led to a wave in synthesis of improved chemically modified tetracyclines (CMT) with deletion of the anti-microbial functional group but enhancing their MMP-directed inhibitory potencies (157). The main mode of action is chelation of Zn^2+^ and Ca^2+^ ions, but also other mechanisms including regulation of gene expression and degradation of MMPs have been proposed (158). One of the most potent and promising compounds is Metastat (Col-3, Incyclinide) which inhibited the expression and the activity of MMP-2 and reduced tumor growth and metastasis formation in pre-clinical tumor models (159). Interestingly, Metastat only minimally reduced tumor growth in the B16 melanoma model. However, the combined treatment modality with RT led to strong tumor growth delay and reduced angiogenesis (64). Four phase I/II clinical trials and pharmacokinetic studies were completed with Metastat in the treatment of patients with advanced solid malignancies, AIDS-related Kaposi’s sarcoma, refractory metastatic cancer, and recurrent high-grade glioma (160–163). Metastat was well tolerated, but due to weak responses, no further clinical trials have been initiated. Notably Metastat was tested on the clinical level only as single treatment modality and not in combination with radio-/chemotherapy.

Interestingly, many other MMP inhibitors entered clinical trials with promising pre-clinical results to fail dramatically beyond Phase II (137, 158). Approved in 2001 for the treatment of chronic periodontitis, the doxycycline hyclate Periostat targeting collagenase activity in the gingival tissue represents the only FDA approved MMP inhibitor (153, 164, 165). Besides this sole success, decades of research have led us to reason that metalloproteinases do not represent suitable targets for cancer treatment (31, 137, 166, 167). Among many others, the major therapeutic challenge lies in the complexity of the protease network “protease web” as MMPs do not only act alone or in linear pathways, but are part of complex and dynamic amplification cascades or inter-regulatory circuits (166). Disease but also non-specific drugs perturb the order, adding higher spatio-temporal complexity to the network.

### Therapeutic Antibodies

Targeting the catalytic domain of enzymes appears as an attractive therapeutic approach. However, these domains are highly conserved amongst different MMPs, leading to off-target effects and tissue toxicity. As MMPs act extracellularly they represent excellent targets for highly specific inhibitory monoclonal antibodies (mAb). Due to their versatile involvement in modulating the tumor microenvironment, inhibitory antibodies against MMP-9, MT1-MMP, and ADAM17 have recently been developed (168–175).

The strong influence of MT1-MMP on the tumor microenvironment renders it an attractive target for therapeutic strategies (90). Therefore, a range of antibodies have been designed that selectively block MT1-MMP, resulting in reduced tumor growth, angiogenesis, and dissemination in ovarian, breast, and melanoma tumor models (169, 172, 176–178). Discovered using phage display technology, DX-2400 blocked MT1-MMP with high potency and reduced tumor burden by inhibiting MMP-2 activation (94, 176). Subsequently, antibody treatment resulted in improved tissue perfusion leading to re-oxygenation of the tumor. This effect could be exploited by combined treatment with radiotherapy leading to additive tumor control in a murine mammary tumor model (94).

The challenge of selectively targeting MMP-9 lies in its structural similarity to MMP-2. The mAb REGA-3G12 solely binds to the catalytic domain of MMP-9, however and despite its strong binding affinity, REGA-3G12 only displays weak inhibitory activity (179, 180). Combining its target specificity with a small molecular MMP inhibitor, gave rise to an antibody-drug conjugate (ADC) consisting of REGA-3G12 and the broad spectrum inhibitor CGS27023A (181). *In vitro*, this ADC could bind to its target with high selectivity, while strongly inhibiting MMP-9 activity. It will be of interest to observe future validation and applications of this elegant approach of combining mAb with a small molecular inhibitor, alone and in combination with systemic chemotherapeutics and radiotherapy.

Selective inhibition of MMP-9 with the two monoclonal antibodies AB0041 and AB0046 reduced symptoms of DSS-induced ulcerative colitis and colorectal tumor burden in murine orthotopic tumor models (168). Given these encouraging results, AB0041 was humanized (GS-5745) towards clinic trials. This makes GS-5745/Andecaliximab the first anti-MMP antibody to currently undergo clinical investigation as monotherapy and as part of a combined treatment modality with chemotherapy (182–184).

Only in the last decade the first promising anti-human ADAM17 antibody D1(A12) was developed (171). Characteristic for this cross-domain antibody is its simultaneous recognition of catalytic as well as noncatalytic regions, acting as steric hindrance and allosteric inhibitor at the same time (171). D1(A12) was shown to bind to ADAM17 in a subnanomolar range (*K_D_* of 0.46 nM), reduced cleavage of ADAM17-specific substrates *in vitro* and *in vivo*, mitigated cell migration, and inhibited tumor growth with suitable pharmacokinetics (185–187). Two other antibodies, A9(B8) and MEDI3622, are currently undergoing pre-clinical investigation and demonstrate anti-tumor effects by inhibiting EGFR-dependent and -independent pathways (170, 174). Characteristic for MEDI3266 is its high site-specificity and target-sensitivity as it recognizes the surface loop sIVa-sIVb β-hairpin on the M-domain, unique for ADAM17 (188). MEDI3266 was shown to inhibit tumor growth of different tumor models. Combined treatment with EGFR-directed cetuximab led to complete tumor regression in the OE21 esophageal xenograft model and others (174). Furthermore, ADAM17-inhibition by MEDI3266 blocked CD16A cleavage from activated NK-cells (see above) and resulted in increased production of IFNγ in the presence of antibody-opsonized tumor cells (189). Several studies have investigated the consequences of CD16A blocking for antibody-dependent cellular cytotoxicity (ADCC), though with contradictory results (124). Hence, the precise ways on how ADAM17-inhibition leads to tumor reduction and the involvement of ADCC remains to determined. Nevertheless, based on their specificity, these ADAM17-directed antibodies represent ideal candidates for a combined treatment modality with RT.

## Discussion

Modern image-guided radiotherapy has reached a level of technical conformity that nowadays requires biological means to further increase the therapeutic window towards improved treatment outcome e.g. as part of combined treatment modalities with highly potent pharmacological agents specifically sensitizing the tumor compartment to ionizing radiation. While radiotherapy combined with classic chemotherapeutic agents e.g. cisplatin for head and neck squamous cell carcinoma became standard clinical practice within the last twenty years, combined treatment with small molecular agents or inhibitory antibodies targeting specific signaling moieties are still considered exceptional. This might be due to the continuous development of new radiotherapeutic treatment regimens, from classic fractionated low dose to hypofractionated and stereotactic single high dose treatment regimens. Indeed, different radiotherapy regimens induce differential biological processes and thus require adaptations, also in the choice of a combined treatment modality. On the other hand, major resistance factors for successful radiotherapy, such as tumor hypoxia, cannot be linked to a specific signal transduction cascade or a defined genetic background, rendering personalized radiochemotherapeutic approaches very difficult. With the exception of immune checkpoint inhibitors targeting specific intercellular signaling moieties and their rapid integration into clinical radioimmunotherapy protocols within the last five years, the combined treatment modality of radiotherapy with molecularly defined targeting agents did not reach maturity.

Thus, the slow progress towards a clinically relevant combined treatment modality of ionizing radiation with inhibitors of metalloproteinases is not an exception and includes additional hurdles. The development of small molecular compounds targeting selected metalloproteinases with sufficient specificity has not been successful so far without inducing limiting toxicities on the clinical level. This might be further restricted by existing redundancies in between different metalloproteinases for relevant substrates and represents an intrinsic challenge even for therapeutic antibodies targeting individual metalloproteinases with highest specificity. On the other hand, such inhibitory antibodies might be accompanied by reduced normal tissue toxicities. Recent advances e.g. with ADAM17-targeting antibodies demonstrate promising results on the preclinical level (see above) and are currently also probed in combination with radiotherapy.

Individual metalloproteinases have a plethora of different substrates thereby co-regulating multiple biological processes, hallmarks of cancer and thus putative intrinsic treatment-induced resistance mechanisms at the same time. Thereby inhibition of individual metalloproteinases might affect at the same time not only the composition of the extracellular matrix but also tumor growth, tumor angiogenesis and immune cell infiltration via reduced shedding of respective bioactive substrates, such as tumor growth and pro-angiogenic factors, chemo- and cytokines. Insofar our knowledge on the role and the complexity of metalloproteinases for tumorigenesis, tumor growth and dissemination is steadily increasing.

**Figure 3:**
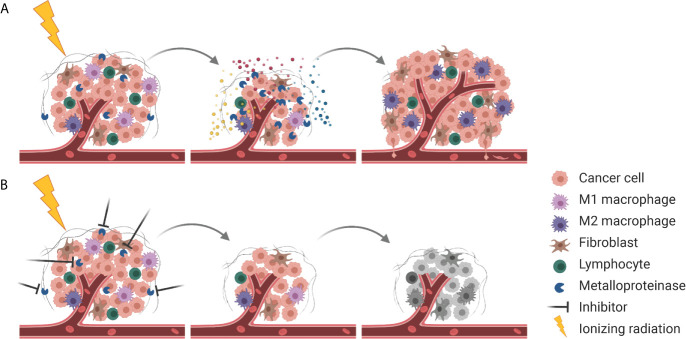
Combining RT with metalloproteinase inhibitors for improved tumor control. **(A)** Next to cell killing and tumor shrinkage, RT activates metalloproteinases that release pro-survival factors (indicated in blue, red, and yellow) into the TME, resulting in tumor cell proliferation, enhanced tumor angiogenesis and pro-tumorigenic immune responses. At the same metalloproteinases disrupt the ECM barrier (gray), enabling tumor cell dissemination. **(B)** Combining RT with inhibition of metalloproteinases mitigates pro-survival signaling and results in more effective tumor cell killing.

Interestingly, many of the aforementioned processes are also triggered by radiotherapy. The insult on the level of DNA is most important for the cytotoxicity of radiotherapy. However, ionizing radiation also affects multiple intra- and inter-cellular processes thereby determining the tumor response to radiotherapy and eventually treatment outcome. Irradiated tumor, stromal and endothelial cells release auto- and paracrine factors in response to radiotherapy-induced DNA damage and radiotherapy-activated intracellular stress-responses, which subsequently modulate the tumor microenvironment and the radiosensitivity of the respective target cells. We currently recognize that these intercellular processes are often mediated via basal and even more so ionizing radiation-induced metalloproteinase activities rendering metalloproteinases to become interesting targets in this context (**Figure 3**). Furthermore, the intratumoral bystander effect induced by inhibition of extracellularly located metalloproteinase activities will conceptually also take advantage of and synergize with locoregionally applied ionizing radiation reaching each individual tumor cell. As such, targeting of specific metalloproteinases in combination with radiotherapy represents a highly promising treatment strategy; however, we still need to identify the best needle in the haystack.

## Author Contributions

VW performed literature search, drafted, and edited the manuscript. MP participated in conceptualization, revised, and finalized the manuscript. All authors contributed to the article and approved the submitted version.

## Funding

This work was support in part by the Vontobel-Stiftung (2018-0167), Krebsforschung Schweiz (KFS3993) and the Swiss National Science Foundation (172885).

## Conflict of Interest

The authors declare that the research was conducted in the absence of any commercial or financial relationships that could be construed as a potential conflict of interest.

## References

[1] Grubbé EH. Priority in the Therapeutic Use of X-Rays. Radiology (1933) 21:156–62. 10.1148/21.2.156

[2] Gianfaldoni S Gianfaldoni R Wollina U Lotti J Tchernev G Lotti T. An Overview on Radiotherapy: From Its History to Its Current Applications in Dermatology. Open Access Maced J Med Sci (2017) 5:521–5. 10.3889/oamjms.2017.122 PMC5535674 28785349

[3] Lawrence EO Livingston MS. The Production of High Speed Light Ions Without the Use of High Voltages. Phys Rev (1932) 40:19–35. 10.1103/PhysRev.40.19

[4] Connell PP Hellman S. Advances in Radiotherapy and Implications for the Next Century: A Historical Perspective. Cancer Res (2009) 69:383–92. 10.1158/0008-5472.CAN-07-6871 19147546

[5] NCRI Clinical and Translational Radiotherapy Research Working Group Harrington KJ Billingham LJ Brunner TB Burnet NG Chan CS. Guidelines for Preclinical and Early Phase Clinical Assessment of Novel Radiosensitisers. Br J Cancer (2011) 105:628–39. 10.1038/bjc.2011.240 PMC3188925 21772330

[6] Delaney G Jacob S Featherstone C Barton M. The Role of Radiotherapy in Cancer Treatment: Estimating Optimal Utilization From a Review of Evidence-Based Clinical Guidelines. Cancer (2005) 104:1129–37. 10.1002/cncr.21324 16080176

[7] Elshaikh M Ljungman M Ten Haken R Lichter AS. Advances in Radiation Oncology. Annu Rev Med (2006) 57:19–31. 10.1146/annurev.med.57.121304.131431 16409134

[8] Sato K Shimokawa T Imai T. Difference in Acquired Radioresistance Induction Between Repeated Photon and Particle Irradiation. Front Oncol (2019) 9:1213. 10.3389/fonc.2019.01213 31799186 PMC6863406

[9] Willers H Azzoli CG Santivasi WL Xia F. Basic Mechanisms of Therapeutic Resistance to Radiation and Chemotherapy in Lung Cancer. Cancer J (2013) 19:200–7. 10.1097/PPO.0b013e318292e4e3 PMC3668666 23708066

[10] Barker HE Paget JTE Khan AA Harrington KJ. The Tumour Microenvironment After Radiotherapy: Mechanisms of Resistance and Recurrence. Nat Rev Cancer (2015) 15:409–25. 10.1038/nrc3958 PMC4896389 26105538

[11] Munshi A Ramesh R. Mitogen-Activated Protein Kinases and Their Role in Radiation Response. Genes Cancer (2013) 4:401–8. 10.1177/1947601913485414 PMC3863336 24349638

[12] Baskar R Dai J Wenlong N Yeo R Yeoh K-W. Biological Response of Cancer Cells to Radiation Treatment. Front Mol Biosci (2014) 1:24. 10.3389/fmolb.2014.00024 25988165 PMC4429645

[13] Marampon F Ciccarelli C Zani BM. Biological Rationale for Targeting MEK/ERK Pathways in Anti-Cancer Therapy and to Potentiate Tumour Responses to Radiation. Int J Mol Sci (2019) 20:2530. 10.3390/ijms20102530 PMC6567863 31126017

[14] Kim W Youn H Kang C Youn B. Inflammation-Induced Radioresistance is Mediated by ROS-dependent Inactivation of Protein Phosphatase 1 in non-Small Cell Lung Cancer Cells. Apoptosis (2015) 20:1242–52. 10.1007/s10495-015-1141-1 26033480

[15] Kim HS Song M-C Kwak IH Park TJ Lim IK. Constitutive Induction of P-Erk1/2 Accompanied by Reduced Activities of Protein Phosphatases 1 and 2A and MKP3 Due to Reactive Oxygen Species During Cellular Senescence. J Biol Chem (2003) 278:37497–510. 10.1074/jbc.M211739200 12840032

[16] Sharma A Bender S Zimmermann M Riesterer O Broggini-Tenzer A Pruschy MN. Secretome Signature Identifies ADAM17 as Novel Target for Radiosensitization of Non-Small Cell Lung Cancer. Clin Cancer Res (2016) 22:4428–39. 10.1158/1078-0432.CCR-15-2449 27076628

[17] Feys L Descamps B Vanhove C Vral A Veldeman L Vermeulen S. Radiation-Induced Lung Damage Promotes Breast Cancer Lung-Metastasis Through CXCR4 Signaling. Oncotarget (2015) 6:26615–32. 10.18632/oncotarget.5666 PMC4694940 26396176

[18] Huxley-Jones J Clarke T-K Beck C Toubaris G Robertson DL Boot-Handford RP. The Evolution of the Vertebrate Metzincins; Insights From Ciona Intestinalis and Danio Rerio. BMC Evol Biol (2007) 7:63. 10.1186/1471-2148-7-63 17439641 PMC1867822

[19] Bode W Gomis-Rüth F-X Stöckler W. Astacins, Serralysins, Snake Venom and Matrix Metalloproteinases Exhibit Identical Zinc-Binding Environments (HEXXHXXGXXH and Met-turn) and Topologies and Should be Grouped Into a Common Family, the ‘Metzincins’. FEBS Lett (1993) 331:134–40. 10.1016/0014-5793(93)80312-I 8405391

[20] Stöcker W Bode W. Structural Features of a Superfamily of Zinc-Endopeptidases: The Metzincins. Curr Opin Struct Biol (1995) 5:383–90. 10.1016/0959-440X(95)80101-4 7583637

[21] Georgiadis D Yiotakis A. Specific Targeting of Metzincin Family Members With Small-Molecule Inhibitors: Progress Toward a Multifarious Challenge. Bioorg Med Chem (2008) 16:8781–94. 10.1016/j.bmc.2008.08.058 18790648

[22] Seals DF. The ADAMs Family of Metalloproteases: Multidomain Proteins With Multiple Functions. Genes Dev (2003) 17:7–30. 10.1101/gad.1039703 12514095

[23] Cui N Hu M Khalil RA. Biochemical and Biological Attributes of Matrix Metalloproteinases. Prog Mol Biol Transl Sci (2017) 147:1–73. 10.1016/bs.pmbts.2017.02.005 28413025 PMC5430303

[24] Van Wart HE Birkedal-Hansen H. The Cysteine Switch: A Principle of Regulation of Metalloproteinase Activity With Potential Applicability to the Entire Matrix Metalloproteinase Gene Family. Proc Natl Acad Sci (1990) 87:5578–82. 10.1073/pnas.87.14.5578 PMC54368 2164689

[25] Ra H-J Parks WC. Control of Matrix Metalloproteinase Catalytic Activity. Matrix Biol (2007) 26:587–96. 10.1016/j.matbio.2007.07.001 PMC2246078 17669641

[26] Jackson HW Defamie V Waterhouse P Khokha R. Timps: Versatile Extracellular Regulators in Cancer. Nat Rev Cancer (2017) 17:38–53. 10.1038/nrc.2016.115 27932800

[27] Nieuwesteeg MA Willson JA Cepeda M Fox MA Damjanovski S. Functional Characterization of Tissue Inhibitor of Metalloproteinase-1 (TIMP-1) N- and C-Terminal Domains During *Xenopus Laevis* Development. Sci World J (2014) 2014:1–10. 10.1155/2014/467907 PMC3925571 24616631

[28] Bode W Fernandez-Catalan C Grams F Gomis-Ruth F-X Nagase H Tschesche H. Insights Into MMP-TIMP Interactions. Ann N Y Acad Sci (1999) 878:73–91. 10.1111/j.1749-6632.1999.tb07675.x 10415721

[29] Zucker S Schmidt CE Dufour A Kaplan RC Park HI Jiang W. ProMMP-2: TIMP-1 Complexes Identified in Plasma of Healthy Individuals. Connect Tissue Res (2009) 50:223–31. 10.1080/03008200802626970 PMC3286656 19637058

[30] Wojtowicz-Praga SM Dickson RB Hawkins MJ. Matrix Metalloproteinase Inhibitors. Invest New Drugs (1997) 15:61–75. 10.1023/A:1005722729132 9195290

[31] Coussens LM. Matrix Metalloproteinase Inhibitors and Cancer–Trials and Tribulations. Science (2002) 295:2387–92. 10.1126/science.1067100 11923519

[32] Mauviel A. Cytokine Regulation of Metalloproteinase Gene Expression. J Cell Biochem (1993) 53:288–95. 10.1002/jcb.240530404 8300745

[33] McDonnell SE Kerr LD Matrisian LM. Epidermal Growth Factor Stimulation of Stromelysin mRNA in Rat Fibroblasts Requires Induction of Proto-Oncogenes C-Fos and C-Jun and Activation of Protein Kinase C. Mol Cell Biol (1990) 10:4284–93. 10.1128/mcb.10.8.4284 PMC360972 2115124

[34] Lee M Ryu CH Chang HW Kim GC Kim SW Kim SY. Radiotherapy-Associated Furin Expression and Tumor Invasiveness in Recurrent Laryngeal Cancer. Anticancer Res (2016) 36:5117–25. 10.21873/anticanres.11081 27798871

[35] Weiss S Peppin G Ortiz X Ragsdale C Test S. Oxidative Autoactivation of Latent Collagenase by Human Neutrophils. Science (1985) 227:747–9. 10.1126/science.2982211 2982211

[36] Yamamoto K Murphy G Troeberg L. Extracellular Regulation of Metalloproteinases. Matrix Biol (2015) 44–46:255–63. 10.1016/j.matbio.2015.02.007 25701651

[37] Webb AH Gao BT Goldsmith ZK Irvine AS Saleh N Lee RP. Inhibition of MMP-2 and MMP-9 Decreases Cellular Migration, and Angiogenesis in In Vitro Models of Retinoblastoma. BMC Cancer (2017) 17:434. 10.1186/s12885-017-3418-y 28633655 PMC5477686

[38] Liu Y Zhang H Yan L Du W Zhang M Chen H. MMP-2 and MMP-9 Contribute to the Angiogenic Effect Produced by Hypoxia/15-HETE in Pulmonary Endothelial Cells. J Mol Cell Cardiol (2018) 121:36–50. 10.1016/j.yjmcc.2018.06.006 29913136

[39] Bruni-Cardoso A Johnson LC Vessella RL Peterson TE Lynch CC. Osteoclast-Derived Matrix Metalloproteinase-9 Directly Affects Angiogenesis in the Prostate Tumor-Bone Microenvironment. Mol Cancer Res (2010) 8:459–70. 10.1158/1541-7786.MCR-09-0445 PMC2946627 20332212

[40] Xu D McKee CM Cao Y Ding Y Kessler BM Muschel RJ. Matrix Metalloproteinase-9 Regulates Tumor Cell Invasion Through Cleavage of Protease Nexin-1. Cancer Res (2010) 70:6988–98. 10.1158/0008-5472.CAN-10-0242 PMC3272441 20736374

[41] Mehner C Hockla A Miller E Ran S Radisky DC Radisky ES. Tumor Cell-Produced Matrix Metalloproteinase 9 (MMP-9) Drives Malignant Progression and Metastasis of Basal-Like Triple Negative Breast Cancer. Oncotarget (2014) 5:2736–49. 10.18632/oncotarget.1932 PMC4058041 24811362

[42] Blanco-Prieto S Barcia-Castro L Páez de la Cadena M Rodríguez-Berrocal FJ Vázquez-Iglesias L Botana-Rial MI. Relevance of Matrix Metalloproteases in non-Small Cell Lung Cancer Diagnosis. BMC Cancer (2017) 17:823. 10.1186/s12885-017-3842-z 29207990 PMC5718060

[43] Li Y Wu T Zhang B Yao Y Yin G. Matrix Metalloproteinase-9 is a Prognostic Marker for Patients With Cervical Cancer. Med Oncol (2012) 29:3394–9. 10.1007/s12032-012-0283-z 22752570

[44] Zhang H Li G Zhang Z Wang S Zhang S. MMP-2 and MMP-9 Gene Polymorphisms Associated With Cervical Cancer Risk. Int J Clin Exp Pathol (2017) 10:11760–5. PMC6966040 31966538

[45] Tian M Cui Y-Z Song G-H Zong M-J Zhou X-Y Chen Y. Proteomic Analysis Identifies MMP-9, DJ-1 and A1BG as Overexpressed Proteins in Pancreatic Juice From Pancreatic Ductal Adenocarcinoma Patients. BMC Cancer (2008) 8:241. 10.1186/1471-2407-8-241 18706098 PMC2528014

[46] Wang J Shi Q Yuan T Song Q Zhang Y Wei Q. Matrix Metalloproteinase 9 (MMP-9) in Osteosarcoma: Review and Meta-Analysis. Clin Chim Acta (2014) 433:225–31. 10.1016/j.cca.2014.03.023 24704305

[47] Yousef EM Tahir MR St-Pierre Y Gaboury LA. MMP-9 Expression Varies According to Molecular Subtypes of Breast Cancer. BMC Cancer (2014) 14:609. 10.1186/1471-2407-14-609 25151367 PMC4150970

[48] Chou CH Teng C-M Tzen K-Y Chang Y-C Chen J-H Cheng JC-H. MMP-9 From Sublethally Irradiated Tumor Promotes Lewis Lung Carcinoma Cell Invasiveness and Pulmonary Metastasis. Oncogene (2012) 31:458–68. 10.1038/onc.2011.240 21706046

[49] Gu Q He Y Ji J Yao Y Shen W Luo J. Hypoxia-Inducible Factor 1α (Hif-1α) and Reactive Oxygen Species (ROS) Mediates Radiation-Induced Invasiveness Through the SDF-1α/CXCR4 Pathway in non-Small Cell Lung Carcinoma Cells. Oncotarget (2015) 6:10893–907. 10.18632/oncotarget.3535 PMC4484427 25843954

[50] Cheng JC-H Chou CH Kuo ML Hsieh C-Y. Radiation-Enhanced Hepatocellular Carcinoma Cell Invasion With MMP-9 Expression Through PI3K/Akt/NF-κB Signal Transduction Pathway. Oncogene (2006) 25:7009–18. 10.1038/sj.onc.1209706 16732316

[51] Ko Y Jin H Lee J Park S Chang K Kang K. Radioresistant Breast Cancer Cells Exhibit Increased Resistance to Chemotherapy and Enhanced Invasive Properties Due to Cancer Stem Cells. Oncol Rep (2018) 40:3752–3762. 10.3892/or.2018.6714 30272295

[52] Ahn G-O Brown JM. Matrix Metalloproteinase-9 is Required for Tumor Vasculogenesis But Not for Angiogenesis: Role of Bone Marrow-Derived Myelomonocytic Cells. Cancer Cell (2008) 13:193–205. 10.1016/j.ccr.2007.11.032 18328424 PMC2967441

[53] Kioi M Vogel H Schultz G Hoffman RM Harsh GR Brown JM. Inhibition of Vasculogenesis, But Not Angiogenesis, Prevents the Recurrence of Glioblastoma After Irradiation in Mice. J Clin Invest (2010) 120:694–705. 10.1172/JCI40283 20179352 PMC2827954

[54] Brown JM. Vasculogenesis: A Crucial Player in the Resistance of Solid Tumours to Radiotherapy. Br J Radiol (2014) 87:20130686. 10.1259/bjr.20130686 24338942 PMC4064599

[55] Ding G Liu Y Liang C. Efficacy of Radiotherapy on Intermediate and Advanced Lung Cancer and its Effect on Dynamic Changes of Serum Vascular Endothelial Growth Factor and Matrix Metalloproteinase−9. Oncol Lett (2018) 16:219–224. 10.3892/ol.2018.8622 29928404 PMC6006182

[56] Kalanxhi E Hektoen HH Meltzer S Dueland S Flatmark K Ree AH. Circulating Proteins in Response to Combined-Modality Therapy in Rectal Cancer Identified by Antibody Array Screening. BMC Cancer (2016) 16:536. 10.1186/s12885-016-2601-x 27461255 PMC4962367

[57] Rieff EA Hendriks T Rutten HJT Nieuwenhuijzen GAP Gosens MJEM van den Brule AJC. Neoadjuvant Radiochemotherapy Increases Matrix Metalloproteinase Activity in Healthy Tissue in Esophageal Cancer Patients. Ann Surg Oncol (2009) 16:1384–9. 10.1245/s10434-009-0365-0 19224281

[58] Xie T Dong B Yan Y Hu G Xu Y. Association Between MMP-2 Expression and Prostate Cancer: A Meta-Analysis. BioMed Rep (2016) 4:241–5. 10.3892/br.2015.553 PMC4734094 26893846

[59] Deng J Chen W Du Y Wang W Zhang G Tang Y. Synergistic Efficacy of Cullin1 and MMP-2 Expressions in Diagnosis and Prognosis of Colorectal Cancer. Cancer Biomark (2017) 19:57–64. 10.3233/CBM-160341 28269751 PMC13020704

[60] Zhai L-L Cai C-Y Wu Y Tang Z-G. Correlation and Prognostic Significance of MMP-2 and TFPI-2 Differential Expression in Pancreatic Carcinoma. Int J Clin Exp Pathol (2015) 8:682–91. PMC4348899 25755762

[61] Nasr M Ayyad SB El-Lamie IKI Mikhail MY. Expression of Matrix Metalloproteinase-2 in Preinvasive and Invasive Carcinoma of the Uterine Cervix. Eur J Gynaecol Oncol (2005) 26:199–202. 15857029

[62] Fang J Shing Y Wiederschain D Yan L Butterfield C Jackson G. Matrix Metalloproteinase-2 is Required for the Switch to the Angiogenic Phenotype in a Tumor Model. Proc Natl Acad Sci (2000) 97:3884–9. 10.1073/pnas.97.8.3884 PMC18111 10760260

[63] Kargiotis O Chetty C Gondi CS Tsung AJ Dinh DH Gujrati M. Adenovirus-Mediated Transfer of siRNA Against MMP-2 mRNA Results in Impaired Invasion and Tumor-Induced Angiogenesis, Induces Apoptosis In Vitro and Inhibits Tumor Growth In Vivo in Glioblastoma. Oncogene (2008) 27:4830–40. 10.1038/onc.2008.122 PMC2574662 18438431

[64] Kaliski A Maggiorella L Cengel KA Mathe D Rouffiac V Opolon P. Angiogenesis and Tumor Growth Inhibition by a Matrix Metalloproteinase Inhibitor Targeting Radiation-Induced Invasion. Mol Cancer Ther (2005) 4:1717–28. 10.1158/1535-7163.MCT-05-0179 16275993

[65] Belotti D Paganoni P Manenti L Garofalo A Marchini S Taraboletti G. Matrix Metalloproteinases (MMP9 and MMP2) Induce the Release of Vascular Endothelial Growth Factor (VEGF) by Ovarian Carcinoma Cells: Implications for Ascites Formation. Cancer Res (2003) 63:5224–9. 14500349

[66] Chetty C Bhoopathi P Rao JS Lakka SS. Inhibition of Matrix Metalloproteinase-2 Enhances Radiosensitivity by Abrogating Radiation-Induced FoxM1-mediated G2/M Arrest in A549 Lung Cancer Cells. Int J Cancer (2009) 124:2468–77. 10.1002/ijc.24209 PMC2663016 19165865

[67] Butkiewicz D Krześniak M Drosik A Giglok M Gdowicz-Kłosok A Kosarewicz A. The *VEGFR2* , *COX-2* and *MMP-2* Polymorphisms are Associated With Clinical Outcome of Patients With Inoperable non-Small Cell Lung Cancer: *VEGFR2*, *COX-2* and *MMP-2* Variants and NSCLC Prognosis. Int J Cancer (2015) 137:2332–42. 10.1002/ijc.29605 25975224

[68] Wild-Bode C Weller M Rimner A Dichgans J Wick W. Sublethal Irradiation Promotes Migration and Invasiveness of Glioma Cells: Implications for Radiotherapy of Human Glioblastoma. Cancer Res (2001) 61:2744–50. 11289157

[69] Mehvar R Gross ME Kreamer RN. Pharmacokinetics of Atenolol Enantiomers in Humans and Rats. J Pharm Sci (1990) 79:881–5. 10.1002/jps.2600791007 2280355

[70] Li H Qiu Z Li F Wang C. The Relationship Between MMP-2 and MMP-9 Expression Levels With Breast Cancer Incidence and Prognosis. Oncol Lett (2017) 14:5865–70. 10.3892/ol.2017.6924 PMC5661385 29113219

[71] Kumar A Collins HM Scholefield JH Watson SA. Increased type-IV Collagenase (MMP-2 and MMP-9) Activity Following Preoperative Radiotherapy in Rectal Cancer. Br J Cancer (2000) 82:960–5. 10.1054/bjoc.1999.1025 PMC2374410 10732772

[72] Chan LW Moses MA Goley E Sproull M Muanza T Coleman CN. Urinary VEGF and MMP Levels As Predictive Markers of 1-Year Progression-Free Survival in Cancer Patients Treated With Radiation Therapy: A Longitudinal Study of Protein Kinetics Throughout Tumor Progression and Therapy. J Clin Oncol (2004) 22:499–506. 10.1200/JCO.2004.07.022 14752073

[73] Smith ER Zurakowski D Saad A Scott RM Moses MA. Urinary Biomarkers Predict Brain Tumor Presence and Response to Therapy. Clin Cancer Res (2008) 14:2378–86. 10.1158/1078-0432.CCR-07-1253 18413828

[74] Waas ET Hendriks T Lomme RMLM Wobbes T. Plasma Levels of Matrix Metalloproteinase-2 and Tissue Inhibitor of Metalloproteinase-1 Correlate With Disease Stage and Survival in Colorectal Cancer Patients. Dis Colon Rectum (2005) 48:700–10. 10.1007/s10350-004-0854-y 15906450

[75] Sotomayor EA Teicher BA Schwartz GN Holden SA Menon K Herman TS. Minocycline in Combination With Chemotherapy or Radiation Therapy In Vitro and In Vivo. Cancer Chemother Pharmacol (1992) 30:377–84. 10.1007/BF00689966 1505076

[76] Stansborough RL Al-Dasooqi N Bateman EH Bowen JM Keefe DMK Logan RM. Matrix Metalloproteinase Expression is Altered in the Small and Large Intestine Following Fractionated Radiation In Vivo. Support Care Cancer (2018) 26:3873–82. 10.1007/s00520-018-4255-5 29754212

[77] Strup-Perrot C Vozenin-Brotons M-C Vandamme M Benderitter M Mathe D. Expression and Activation of MMP -2, -3, -9, -14 are Induced in Rat Colon After Abdominal X-Irradiation. Scand J Gastroenterol (2006) 41:60–70. 10.1080/00365520510023963 16373278

[78] Angenete E Öresland T Falk P Breimer M Hultborn R Ivarsson M-L. Preoperative Radiotherapy and Extracellular Matrix Remodeling in Rectal Mucosa and Tumour Matrix Metalloproteinases and Plasminogen Components. Acta Oncol (2009) 48:1144–51. 10.3109/02841860903150510 19863222

[79] Angenete E Langenskiöld M Falk P Ivarsson M-L. Matrix Metalloproteinases in Rectal Mucosa, Tumour and Plasma: Response After Preoperative Irradiation. Int J Colorectal Dis (2007) 22:667–74. 10.1007/s00384-006-0225-3 17109104

[80] Kumar A Collins H Van Tam J Scholefield JH Watson SA. Effect of Preoperative Radiotherapy on Matrilysin Gene Expression in Rectal Cancer. Eur J Cancer (2002) 38:505–10. 10.1016/S0959-8049(01)00392-6 11872342

[81] Zhang W Li Y Yang L Zhou B Chen K-L Meng W-J. Knockdown of MMP-7 Inhibits Cell Proliferation and Enhances Sensitivity to 5-Fluorouracil and X-ray Irradiation in Colon Cancer Cells. Clin Exp Med (2014) 14:99–106. 10.1007/s10238-012-0212-7 23086188

[82] Stene C Polistena A Gaber A Nodin B Ottochian B Adawi D. MMP7 Modulation by Short- and Long-term Radiotherapy in Patients With Rectal Cancer. In Vivo (2018) 32:133–8. 10.21873/invivo.11215 PMC5892640 29275310

[83] Singh SS Bhatt MLB Kushwaha VS Singh A Kumar R Gupta R. Role of Matrix Metalloproteinase 13 Gene Expression in the Evaluation of Radiation Response in Oral Squamous Cell Carcinoma. J Carcinog (2017) 16:2. 10.4103/jcar.JCar_5_16 28694741 PMC5490341

[84] Wang J Li Y Wang J Li C Yu K Wang Q. Increased Expression of Matrix Metalloproteinase-13 in Glioma is Associated With Poor Overall Survival of Patients. Med Oncol (2012) 29:2432–7. 10.1007/s12032-012-0181-4 22351249

[85] Luukkaa M Vihinen P Kronqvist P Vahlberg T Pyrhönen S Kähäri V-M. Association Between High Collagenase-3 Expression Levels and Poor Prognosis in Patients With Head and Neck Cancer. Head Neck (2006) 28:225–34. 10.1002/hed.20322 16302191

[86] Quintero-Fabián S Arreola R Becerril-Villanueva E Torres-Romero JC Arana-Argáez V Lara-Riegos J. Role of Matrix Metalloproteinases in Angiogenesis and Cancer. Front Oncol (2019) 9:1370. 10.3389/fonc.2019.01370 31921634 PMC6915110

[87] Seiki M. Membrane-Type 1 Matrix Metalloproteinase: A Key Enzyme for Tumor Invasion. Cancer Lett (2003) 194:1–11. 10.1016/S0304-3835(02)00699-7 12706853

[88] Liu G Atteridge CL Wang X Lundgren AD Wu JD. Cutting Edge: The Membrane Type Matrix Metalloproteinase MMP14 Mediates Constitutive Shedding of MHC Class I Chain-Related Molecule A Independent of A Disintegrin and Metalloproteinases. J Immunol (2010) 184:3346–50. 10.4049/jimmunol.0903789 PMC3191873 20208009

[89] Thakur V Bedogni B. The Membrane Tethered Matrix Metalloproteinase MT1-MMP At the Forefront of Melanoma Cell Invasion and Metastasis. Pharmacol Res (2016) 111:17–22. 10.1016/j.phrs.2016.05.019 27221755

[90] Tomari T Koshikawa N Uematsu T Shinkawa T Hoshino D Egawa N. High Throughput Analysis of Proteins Associating With a Proinvasive MT1-MMP in Human Malignant Melanoma A375 Cells. Cancer Sci (2009) 100:1284–90. 10.1111/j.1349-7006.2009.01173.x PMC11158561 19432894

[91] Kajita M Itoh Y Chiba T Mori H Okada A Kinoh H. Membrane-Type 1 Matrix Metalloproteinase Cleaves Cd44 and Promotes Cell Migration. J Cell Biol (2001) 153:893–904. 10.1083/jcb.153.5.893 11381077 PMC2174329

[92] Paquette B Therriault H Desmarais G Wagner R Royer R Bujold R. Radiation-Enhancement of MDA-MB-231 Breast Cancer Cell Invasion Prevented by a Cyclooxygenase-2 Inhibitor. Br J Cancer (2011) 105:534–41. 10.1038/bjc.2011.260 PMC3170962 21792195

[93] Bouchard G Therriault H Geha S Bujold R Saucier C Paquette B. Radiation-Induced Lung Metastasis Development is MT1-MMP-dependent in a Triple-Negative Breast Cancer Mouse Model. Br J Cancer (2017) 116:479–88. 10.1038/bjc.2016.448 PMC5318978 28103615

[94] Ager EI Kozin SV Kirkpatrick ND Seano G Kodack DP Askoxylakis V. Blockade of MMP14 Activity in Murine Breast Carcinomas: Implications for Macrophages, Vessels, and Radiotherapy. J Natl Cancer Inst (2015) 107:djv017. 10.1093/jnci/djv017 25710962 PMC4402365

[95] Mu D Cambier S Fjellbirkeland L Baron JL Munger JS Kawakatsu H. The Integrin αvβ8 Mediates Epithelial Homeostasis Through MT1-MMP–dependent Activation of TGF-β1. J Cell Biol (2002) 157:493–507. 10.1083/jcb.200109100 11970960 PMC2173277

[96] Gray LH Conger AD Ebert M Hornsey S Scott OCA. The Concentration of Oxygen Dissolved in Tissues At the Time of Irradiation as a Factor in Radiotherapy. Br J Radiol (1953) 26:638–48. 10.1259/0007-1285-26-312-638 13106296

[97] Thakur V Zhang K Savadelis A Zmina P Aguila B Welford SM. The Membrane Tethered Matrix Metalloproteinase MT1-MMP Triggers an Outside-in DNA Damage Response That Impacts Chemo- and Radiotherapy Responses of Breast Cancer. Cancer Lett (2019) 443:115–24. 10.1016/j.canlet.2018.11.031 PMC7185872 30502358

[98] Furmanova-Hollenstein P Broggini-Tenzer A Eggel M Millard A-L Pruschy M. The Microtubule Stabilizer Patupilone Counteracts Ionizing Radiation-Induced Matrix Metalloproteinase Activity and Tumor Cell Invasion. Radiat Oncol Lond Engl (2013) 8:105. 10.1186/1748-717X-8-105 PMC3661365 23631818

[99] Giaccone G. EGFR Point Mutation Confers Resistance to Gefitinib in a Patient With non-Small-Cell Lung Cancer. Nat Clin Pract Oncol (2005) 2:296–7. 10.1038/ncponc0200 16264986

[100] Bonner JA Harari PM Giralt J Azarnia N Shin DM Cohen RB. Radiotherapy Plus Cetuximab for Squamous-Cell Carcinoma of the Head and Neck. N Engl J Med (2006) 354:567–78. 10.1056/NEJMoa053422 16467544

[101] Bradley JD Paulus R Komaki R Masters G Blumenschein G Schild S. Standard-Dose Versus High-Dose Conformal Radiotherapy With Concurrent and Consolidation Carboplatin Plus Paclitaxel With or Without Cetuximab for Patients With Stage IIIA or IIIB non-Small-Cell Lung Cancer (RTOG 0617): A Randomised, Two-by-Two Factorial Phase 3 Study. Lancet Oncol (2015) 16:187–99. 10.1016/S1470-2045(14)71207-0 PMC4419359 25601342

[102] Mochizuki S Okada Y. Adams in Cancer Cell Proliferation and Progression. Cancer Sci (2007) 98:621–8. 10.1111/j.1349-7006.2007.00434.x PMC11160018 17355265

[103] Kataoka H. EGFR Ligands and Their Signaling Scissors, ADAMs, as New Molecular Targets for Anticancer Treatments. J Dermatol Sci (2009) 56:148–53. 10.1016/j.jdermsci.2009.10.002 19896805

[104] Chang L Graham P Hao J Ni J Deng J Bucci J. Cancer Stem Cells and Signaling Pathways in Radioresistance. Oncotarget (2016) 7:11002–17. 10.18632/oncotarget.6760 PMC4905454 26716904

[105] Hong SW Hur W Choi JE Kim J-H Hwang D Yoon SK. Role of ADAM17 in Invasion and Migration of CD133-expressing Liver Cancer Stem Cells After Irradiation. Oncotarget (2016) 7:23482–97. 10.18632/oncotarget.8112 PMC5029641 26993601

[106] Mueller AC Piper M Goodspeed A Bhuvane S Williams JS Bhatia S. Induction of ADAM10 by RT Drives Fibrosis, Resistance, and EMT in Pancreatic Cancer. Cancer Res (2021), canres.CAN–20-3892-A.2020. 10.1158/0008-5472.CAN-20-3892 PMC8260469 33526513

[107] Kabacik S Raj K. Ionising Radiation Increases Permeability of Endothelium Through ADAM10-mediated Cleavage of VE-Cadherin. Oncotarget (2017) 8:82049–63. 10.18632/oncotarget.18282 PMC5669869 29137243

[108] Kouam PN Rezniczek GA Adamietz IA Bühler H. Ionizing Radiation Increases the Endothelial Permeability and the Transendothelial Migration of Tumor Cells Through ADAM10-activation and Subsequent Degradation of VE-Cadherin. BMC Cancer (2019) 19:958. 10.1186/s12885-019-6219-7 31619190 PMC6794838

[109] Kim D Lee M Kim E. Involvement of Klotho, TNF−&alpha; and ADAMs in Radiation−Induced Senescence of Renal Epithelial Cells. Mol Med Rep (2020) 23:1–1. 10.3892/mmr.2020.11660 33179086 PMC7673348

[110] Masoud GN Li W. HIF-1α Pathway: Role, Regulation and Intervention for Cancer Therapy. Acta Pharm Sin B (2015) 5:378–89. 10.1016/j.apsb.2015.05.007 PMC4629436 26579469

[111] Choi JY Jang YS Min SY Song JY. Overexpression of MMP-9 and HIF-1α in Breast Cancer Cells Under Hypoxic Conditions. J Breast Cancer (2011) 14:88. 10.4048/jbc.2011.14.2.88 21847402 PMC3148536

[112] Shan Y You B Shi S Shi W Zhang Z Zhang Q. Hypoxia-Induced Matrix Metalloproteinase-13 Expression in Exosomes From Nasopharyngeal Carcinoma Enhances Metastases. Cell Death Dis (2018) 9:382. 10.1038/s41419-018-0425-0 29515112 PMC5841433

[113] Shin DH Dier U Melendez JA Hempel N. Regulation of MMP-1 Expression in Response to Hypoxia is Dependent on the Intracellular Redox Status of Metastatic Bladder Cancer Cells. Biochim Biophys Acta (2015) 1852:2593–602. 10.1016/j.bbadis.2015.09.001 PMC4615546 26343184

[114] Chen J-Y Lin C-H Chen B-C. Hypoxia-Induced ADAM 17 Expression is Mediated by RSK1-dependent C/EBPβ Activation in Human Lung Fibroblasts. Mol Immunol (2017) 88:155–63. 10.1016/j.molimm.2017.06.029 28646679

[115] Noda K Ishida S Shinoda H Koto T Aoki T Tsubota K. Hypoxia Induces the Expression of Membrane-Type 1 Matrix Metalloproteinase in Retinal Glial Cells. Invest Ophthalmol Vis Sci (2005) 46:3817. 10.1167/iovs.04-1528 16186369

[116] Barsoum IB Hamilton TK Li X Cotechini T Miles EA Siemens DR. Hypoxia Induces Escape From Innate Immunity in Cancer Cells Via Increased Expression of ADAM10: Role of Nitric Oxide. Cancer Res (2011) 71:7433–41. 10.1158/0008-5472.CAN-11-2104 22006996

[117] Charbonneau M Harper K Grondin F Pelmus M McDonald PP Dubois CM. Hypoxia-Inducible Factor Mediates Hypoxic and Tumor Necrosis Factor α-Induced Increases in Tumor Necrosis Factor-α Converting Enzyme/Adam17 Expression by Synovial Cells. J Biol Chem (2007) 282:33714–24. 10.1074/jbc.M704041200 17884817

[118] Pastorekova S Gillies RJ. The Role of Carbonic Anhydrase IX in Cancer Development: Links to Hypoxia, Acidosis, and Beyond. Cancer Metastasis Rev (2019) 38:65–77. 10.1007/s10555-019-09799-0 31076951 PMC6647366

[119] Zatovicova M Sedlakova O Svastova E Ohradanova A Ciampor F Arribas J. Ectodomain Shedding of the Hypoxia-Induced Carbonic Anhydrase IX is a Metalloprotease-Dependent Process Regulated by TACE/ADAM17. Br J Cancer (2005) 93:1267–76. 10.1038/sj.bjc.6602861 PMC2361518 16278664

[120] Kajanova I Zatovicova M Jelenska L Sedlakova O Barathova M Csaderova L. Impairment of Carbonic Anhydrase IX Ectodomain Cleavage Reinforces Tumorigenic and Metastatic Phenotype of Cancer Cells. Br J Cancer (2020) 122:1590–603. 10.1038/s41416-020-0804-z PMC7250822 32210366

[121] Lambrecht BN Vanderkerken M Hammad H. The Emerging Role of ADAM Metalloproteinases in Immunity. Nat Rev Immunol (2018) 18:745–58. 10.1038/s41577-018-0068-5 30242265

[122] Romee R Lenvik T Wang Y Walcheck B Verneris MR Miller JS. ADAM17, a Novel Metalloproteinase, Mediates CD16 and CD62L Shedding in Human Nk Cells and Modulates IFNγ Responses. Blood (2011) 118:2184–4. 10.1182/blood.V118.21.2184.2184

[123] Lajoie L Congy-Jolivet N Bolzec A Gouilleux-Gruart V Sicard E Sung HC. ADAM17-Mediated Shedding of FcγRIIIA on Human NK Cells: Identification of the Cleavage Site and Relationship With Activation. J Immunol (2014) 192:741–51. 10.4049/jimmunol.1301024 24337742

[124] Wu J Mishra HK Walcheck B. Role of ADAM17 as a Regulatory Checkpoint of CD16A in NK Cells and as a Potential Target for Cancer Immunotherapy. J Leukoc Biol (2019) 105:1297–303. 10.1002/JLB.2MR1218-501R PMC6792391 30786043

[125] Waldhauer I Goehlsdorf D Gieseke F Weinschenk T Wittenbrink M Ludwig A. Tumor-Associated MICA is Shed by ADAM Proteases. Cancer Res (2008) 68:6368–76. 10.1158/0008-5472.CAN-07-6768 18676862

[126] Boutet P Agüera-González S Atkinson S Pennington CJ Edwards DR Murphy G. Cutting Edge: The Metalloproteinase ADAM17/TNF-alpha-converting Enzyme Regulates Proteolytic Shedding of the MHC Class I-related Chain B Protein. J Immunol Baltim Md 1950 (2009) 182:49–53. 10.4049/jimmunol.182.1.49 19109134

[127] Mohammed RN Wehenkel SC Galkina EV Yates E-K Preece G Newman A. ADAM17-Dependent Proteolysis of L-selectin Promotes Early Clonal Expansion of Cytotoxic T Cells. Sci Rep (2019) 9:5487. 10.1038/s41598-019-41811-z 30940840 PMC6445073

[128] Orme JJ Jazieh KA Xie T Harrington S Liu X Ball M. ADAM10 and ADAM17 Cleave PD-L1 to Mediate PD-(L)1 Inhibitor Resistance. Oncoimmunology (2020) 9:1744980. 10.1080/2162402X.2020.1744980 32363112 PMC7185206

[129] Romero Y Wise R Zolkiewska A. Proteolytic Processing of PD-L1 by ADAM Proteases in Breast Cancer Cells. Cancer Immunol Immunother (2020) 69:43–55. 10.1007/s00262-019-02437-2 31796994 PMC6952561

[130] Sørensen BS Horsman MR. Tumor Hypoxia: Impact on Radiation Therapy and Molecular Pathways. Front Oncol (2020) 10:562. 10.3389/fonc.2020.00562 32373534 PMC7186437

[131] Colliez F Gallez B Jordan BF. Assessing Tumor Oxygenation for Predicting Outcome in Radiation Oncology: A Review of Studies Correlating Tumor Hypoxic Status and Outcome in the Preclinical and Clinical Settings. Front Oncol (2017) 7:10. 10.3389/fonc.2017.00010 28180110 PMC5263142

[132] Noman MZ Hasmim M Messai Y Terry S Kieda C Janji B. Hypoxia: A Key Player in Antitumor Immune Response. A Review in the Theme: Cellular Responses to Hypoxia. Am J Physiol Cell Physiol (2015) 309:C569–79. 10.1152/ajpcell.00207.2015 PMC4628936 26310815

[133] Eckert F Zwirner K Boeke S Thorwarth D Zips D Huber SM. Rationale for Combining Radiotherapy and Immune Checkpoint Inhibition for Patients With Hypoxic Tumors. Front Immunol (2019) 10:407. 10.3389/fimmu.2019.00407 30930892 PMC6423917

[134] Whittaker M Floyd CD Brown P Gearing AJH. Design and Therapeutic Application of Matrix Metalloproteinase Inhibitors. Chem Rev (1999) 99:2735–76. 10.1021/cr9804543 11749499

[135] Rothenberg ML Nelson AR Hande KR. New Drugs on the Horizon: Matrix Metalloproteinase Inhibitors. Stem Cells (1999) 17:237–40. 10.1002/stem.170237 10437989

[136] Egeblad M Werb Z. New Functions for the Matrix Metalloproteinases in Cancer Progression. Nat Rev Cancer (2002) 2:161–74. 10.1038/nrc745 11990853

[137] Fields GB. The Rebirth of Matrix Metalloproteinase Inhibitors: Moving Beyond the Dogma. Cells (2019) 8:984. 10.3390/cells8090984 PMC6769477 31461880

[138] Jacobsen JA Major Jourden JL Miller MT Cohen SM. To Bind Zinc or Not to Bind Zinc: An Examination of Innovative Approaches to Improved Metalloproteinase Inhibition. Biochim Biophys Acta BBA - Mol Cell Res (2010) 1803:72–94. 10.1016/j.bbamcr.2009.08.006 19712708

[139] Bissett D O’Byrne KJ von Pawel J Gatzemeier U Price A Nicolson M. Phase III Study of Matrix Metalloproteinase Inhibitor Prinomastat in Non–Small-Cell Lung Cancer. J Clin Oncol (2005) 23:842–9. 10.1200/JCO.2005.03.170 15681529

[140] Shu C Zhou H Afsharvand M Duan L Zhang H Noveck R. Pharmacokinetic-Pharmacodynamic Modeling of Apratastat: A Population-Based Approach. J Clin Pharmacol (2011) 51:472–81. 10.1177/0091270010372389 21059888

[141] Moss ML Minond D. Recent Advances in ADAM17 Research: A Promising Target for Cancer and Inflammation. Mediators Inflamm (2017) 2017:1–21. 10.1155/2017/9673537 PMC5688260 29230082

[142] Zhou B-BS Peyton M He B Liu C Girard L Caudler E. Targeting ADAM-mediated Ligand Cleavage to Inhibit HER3 and EGFR Pathways in non-Small Cell Lung Cancer. Cancer Cell (2006) 10:39–50. 10.1016/j.ccr.2006.05.024 16843264 PMC4451119

[143] Fridman JS Caulder E Hansbury M Liu X Yang G Wang Q. Selective Inhibition of ADAM Metalloproteases as a Novel Approach for Modulating ErbB Pathways in Cancer. Clin Cancer Res (2007) 13:1892–902. 10.1158/1078-0432.CCR-06-2116 17363546

[144] Witters L Scherle P Friedman S Fridman J Caulder E Newton R. Synergistic Inhibition With a Dual Epidermal Growth Factor Receptor/HER-2/neu Tyrosine Kinase Inhibitor and a Disintegrin and Metalloprotease Inhibitor. Cancer Res (2008) 68:7083–9. 10.1158/0008-5472.CAN-08-0739 18757423

[145] Newton RC Bradley EC Levy RS Doval D Bondarde S Sahoo TP. Clinical Benefit of INCB7839, a Potent and Selective ADAM Inhibitor, in Combination With Trastuzumab in Patients With Metastatic HER2+ Breast Cancer. J Clin Oncol (2010) 28:3025–5. 10.1200/jco.2010.28.15_suppl.3025

[146] Feldinger K Generali D Kramer-Marek G Gijsen M Ng TB Wong JH. ADAM10 Mediates Trastuzumab Resistance and is Correlated With Survival in HER2 Positive Breast Cancer. Oncotarget (2014) 5:6633–46. 10.18632/oncotarget.1955 PMC4196152 24952873

[147] Van Schaeybroeck S Karaiskou-McCaul A Kelly D Longley D Galligan L Van Cutsem E. Epidermal Growth Factor Receptor Activity Determines Response of Colorectal Cancer Cells to Gefitinib Alone and in Combination With Chemotherapy. Clin Cancer Res (2005) 11:7480–9. 10.1158/1078-0432.CCR-05-0328 16243822

[148] Kyula JN Van Schaeybroeck S Doherty J Fenning CS Longley DB Johnston PG. Chemotherapy-Induced Activation of ADAM-17: A Novel Mechanism of Drug Resistance in Colorectal Cancer. Clin Cancer Res (2010) 16:3378–89. 10.1158/1078-0432.CCR-10-0014 PMC2896550 20570921

[149] Wolpert F Tritschler I Steinle A Weller M Eisele G. A Disintegrin and Metalloproteinases 10 and 17 Modulate the Immunogenicity of Glioblastoma-Initiating Cells. Neuro Oncol (2014) 16:382–91. 10.1093/neuonc/not232 PMC3922520 24327582

[150] Gingras D Boivin D Deckers C Gendron S Barthomeuf C Béliveau R. Neovastat–a Novel Antiangiogenic Drug for Cancer Therapy. Anticancer Drugs (2003) 14:91–6. 10.1097/00001813-200302000-00001 12569294

[151] Dupont E Falardeau P Mousa SA Dimitriadou V Pepin M-C Wang T. Antiangiogenic and Antimetastatic Properties of Neovastat (AE-941), an Orally Active Extract Derived From Cartilage Tissue. Clin Exp Metastasis (2002) 19:145–53. 10.1023/a:1014546909573 11964078

[152] Lu C Lee JJ Komaki R Herbst RS Feng L Evans WK. Chemoradiotherapy With or Without AE-941 in Stage III Non-Small Cell Lung Cancer: A Randomized Phase III Trial. J Natl Cancer Inst (2010) 102:859–65. 10.1093/jnci/djq179 PMC2902826 20505152

[153] Golub LM Ciancio S Ramamurthy NS Leung M McNamara TF. Low-Dose Doxycycline Therapy: Effect on Gingival and Crevicular Fluid Collagenase Activity in Humans. J Periodontal Res (1990) 25:321–30. 10.1111/j.1600-0765.1990.tb00923.x 2177499

[154] Fife RS Sledge GW. Effects of Doxycycline on In Vitro Growth, Migration, and Gelatinase Activity of Breast Carcinoma Cells. J Lab Clin Med (1995) 125:407–11. 7897308

[155] van den Bogert C Dontje BH Holtrop M Melis TE Romijn JC van Dongen JW. Arrest of the Proliferation of Renal and Prostate Carcinomas of Human Origin by Inhibition of Mitochondrial Protein Synthesis. Cancer Res (1986) 46:3283–9. 3011245

[156] Fife RS Rougraff BT Proctor C Sledge GW. Inhibition of Proliferation and Induction of Apoptosis by Doxycycline in Cultured Human Osteosarcoma Cells. J Lab Clin Med (1997) 130:530–4. 10.1016/S0022-2143(97)90130-X 9390641

[157] Yu Z Leung MK Ramamurthy NS McNamara TF Golub LM. HPLC Determination of a Chemically Modified Nonantimicrobial Tetracycline: Biological Implications. Biochem Med Metab Biol (1992) 47:10–20. 10.1016/0885-4505(92)90003-H 1314064

[158] Hidalgo M Eckhardt SG. Development of Matrix Metalloproteinase Inhibitors in Cancer Therapy. J Natl Cancer Inst (2001) 93:178–93. 10.1093/jnci/93.3.178 11158186

[159] Acharya MR Venitz J Figg WD Sparreboom A. Chemically Modified Tetracyclines as Inhibitors of Matrix Metalloproteinases. Drug Resist Updat (2004) 7:195–208. 10.1016/j.drup.2004.04.002 15296861

[160] Syed S Takimoto C Hidalgo M Rizzo J Kuhn JG Hammond LA. A Phase I and Pharmacokinetic Study of Col-3 (Metastat), an Oral Tetracycline Derivative With Potent Matrix Metalloproteinase and Antitumor Properties. Clin Cancer Res (2004) 10:6512–21. 10.1158/1078-0432.CCR-04-0804 15475438

[161] Cianfrocca M Cooley TP Lee JY Rudek MA Scadden DT Ratner L. Matrix Metalloproteinase Inhibitor COL-3 in the Treatment of AIDS-Related Kaposi’s Sarcoma: A Phase I AIDS Malignancy Consortium Study. J Clin Oncol (2002) 20:153–9. 10.1200/JCO.2002.20.1.153 11773164

[162] Rudek MA Figg WD Dyer V Dahut W Turner ML Steinberg SM. Phase I Clinical Trial of Oral COL-3, a Matrix Metalloproteinase Inhibitor, in Patients With Refractory Metastatic Cancer. J Clin Oncol (2001) 19:584–92. 10.1200/JCO.2001.19.2.584 11208854

[163] Rudek MA New P Mikkelsen T Phuphanich S Alavi JB Nabors LB. Phase I and Pharmacokinetic Study of COL-3 in Patients With Recurrent High-Grade Gliomas. J Neurooncol (2011) 105:375–81. 10.1007/s11060-011-0602-9 PMC3197967 21547395

[164] Golub LM Sorsa T Lee HM Ciancio S Sorbi D Ramamurthy NS. Doxycycline Inhibits Neutrophil (PMN)-Type Matrix Metalloproteinases in Human Adult Periodontitis Gingiva. J Clin Periodontol (1995) 22:100–9. 10.1111/j.1600-051x.1995.tb00120.x 7775665

[165] Periostat (Doxycycline 20mg). Br Dent J (2006) 200:115–5. 10.1038/sj.bdj.4813235

[166] Overall CM Kleifeld O. Validating Matrix Metalloproteinases as Drug Targets and Anti-Targets for Cancer Therapy. Nat Rev Cancer (2006) 6:227–39. 10.1038/nrc1821 16498445

[167] Overall CM López-Otín C. Strategies for MMP Inhibition in Cancer: Innovations for the Post-Trial Era. Nat Rev Cancer (2002) 2:657–72. 10.1038/nrc884 12209155

[168] Marshall DC Lyman SK McCauley S Kovalenko M Spangler R Liu C. Selective Allosteric Inhibition of MMP9 is Efficacious in Preclinical Models of Ulcerative Colitis and Colorectal Cancer. PloS One (2015) 10:e0127063. 10.1371/journal.pone.0127063 25961845 PMC4427291

[169] Botkjaer KA Kwok HF Terp MG Karatt-Vellatt A Santamaria S McCafferty J. Development of a Specific Affinity-Matured Exosite Inhibitor to MT1-MMP That Efficiently Inhibits Tumor Cell Invasion *In Vitro* and Metastasis *In Vivo* . Oncotarget (2016) 7:16773–92. 10.18632/oncotarget.7780 PMC4941350 26934448

[170] Yang Z Chan KI Kwok HF Tam KY. Novel Therapeutic Anti-ADAM17 Antibody A9(B8) Enhances EGFR-TKI–Mediated Anticancer Activity in NSCLC. Transl Oncol (2019) 12:1516–24. 10.1016/j.tranon.2019.08.003 PMC6717059 31450127

[171] Tape CJ Willems SH Dombernowsky SL Stanley PL Fogarasi M Ouwehand W. Cross-Domain Inhibition of TACE Ectodomain. Proc Natl Acad Sci (2011) 108:5578–83. 10.1073/pnas.1017067108 PMC3078358 21415364

[172] Lopez T Nam DH Kaihara E Mustafa Z Ge X. Identification of Highly Selective MMP-14 Inhibitory Fabs by Deep Sequencing: Protease Inhibitory mAbs Discovered by Deep Sequencing. Biotechnol Bioeng (2017) 114:1140–50. 10.1002/bit.26248 PMC5398909 28090632

[173] Nam DH Fang K Rodriguez C Lopez T Ge X. Generation of Inhibitory Monoclonal Antibodies Targeting Matrix metalloproteinase-14 by Motif Grafting and CDR Optimization. Protein Eng Des Sel (2017) 30:113–8. 10.1093/protein/gzw070 PMC6283398 27986919

[174] Rios-Doria J Sabol D Chesebrough J Stewart D Xu L Tammali R. A Monoclonal Antibody to ADAM17 Inhibits Tumor Growth by Inhibiting EGFR and Non–EGFR-Mediated Pathways. Mol Cancer Ther (2015) 14:1637–49. 10.1158/1535-7163.MCT-14-1040 25948294

[175] Fischer T Riedl R. Inhibitory Antibodies Designed for Matrix Metalloproteinase Modulation. Molecules (2019) 24:2265. 10.3390/molecules24122265 PMC6631688 31216704

[176] Devy L Huang L Naa L Yanamandra N Pieters H Frans N. Selective Inhibition of Matrix Metalloproteinase-14 Blocks Tumor Growth, Invasion, and Angiogenesis. Cancer Res (2009) 69:1517–26. 10.1158/0008-5472.CAN-08-3255 19208838

[177] Remacle AG Cieplak P Nam DH Shiryaev SA Ge X Strongin AY. Selective Function-Blocking Monoclonal Human Antibody Highlights the Important Role of Membrane Type-1 Matrix Metalloproteinase (MT1-MMP) in Metastasis. Oncotarget (2017) 8:2781–99. 10.18632/oncotarget.13157 PMC5356841 27835863

[178] Kaimal R Aljumaily R Tressel SL Pradhan RV Covic L Kuliopulos A. Selective Blockade of Matrix Metalloprotease-14 With a Monoclonal Antibody Abrogates Invasion, Angiogenesis, and Tumor Growth in Ovarian Cancer. Cancer Res (2013) 73:2457–67. 10.1158/0008-5472.CAN-12-1426 PMC3966518 23423981

[179] Paemen L Martens E Masure S Opdenakker G. Monoclonal Antibodies Specific for Natural Human Neutrophil Gelatinase B Used for Affinity Purification, Quantitation by Two-Site ELISA and Inhibition of Enzymatic Activity. Eur J Biochem (1995) 234:759–65. 10.1111/j.1432-1033.1995.759_a.x 8575432

[180] Martens E Leyssen A Van Aelst I Fiten P Piccard H Hu J. A Monoclonal Antibody Inhibits Gelatinase B/MMP-9 by Selective Binding to Part of the Catalytic Domain and Not to the Fibronectin or Zinc Binding Domains. Biochim Biophys Acta BBA - Gen Subj (2007) 1770:178–86. 10.1016/j.bbagen.2006.10.012 17137715

[181] Love EA Sattikar A Cook H Gillen K Large JM Patel S. Developing an Antibody–Drug Conjugate Approach to Selective Inhibition of an Extracellular Protein. ChemBioChem (2019) 20:754–8. 10.1002/cbic.201800623 PMC6582441 30507063

[182] Shah MA Metges J-P Cunningham D Shiu K-K Wyrwicz L Thai D. A Phase II, Open-Label, Randomized Study to Evaluate the Efficacy and Safety of Andecaliximab Combined With Nivolumab Versus Nivolumab Alone in Subjects With Unresectable or Recurrent Gastric or Gastroesophageal Junction Adenocarcinoma. J Clin Oncol (2019) 37:75–5. 10.1200/JCO.2019.37.4_suppl.75

[183] Bendell JC Starodub A Huang X Maltzman JD Wainberg ZA Shah MA. A Phase 3 Randomized, Double-Blind, Placebo-Controlled Study to Evaluate the Efficacy and Safety of GS-5745 Combined With mFOLFOX6 as First-Line Treatment in Patients With Advanced Gastric or Gastroesophageal Junction Adenocarcinoma. J Clin Oncol (2017) 35:TPS4139–TPS4139. 10.1200/JCO.2017.35.15_suppl.TPS4139

[184] Shah MA Starodub A Sharma S Berlin J Patel M Wainberg ZA. Andecaliximab/GS-5745 Alone and Combined With mFOLFOX6 in Advanced Gastric and Gastroesophageal Junction Adenocarcinoma: Results From a Phase I Study. Clin Cancer Res (2018) 24:3829–37. 10.1158/1078-0432.CCR-17-2469 PMC7496223 29691300

[185] Richards FM Tape CJ Jodrell DI Murphy G. Anti-Tumour Effects of a Specific Anti-ADAM17 Antibody in an Ovarian Cancer Model In Vivo. PloS One (2012) 7:e40597. 10.1371/journal.pone.0040597 22792380 PMC3394719

[186] Caiazza F McGowan PM Mullooly M Murray A Synnott N O’Donovan N. Targeting ADAM-17 With an Inhibitory Monoclonal Antibody has Antitumour Effects in Triple-Negative Breast Cancer Cells. Br J Cancer (2015) 112:1895–903. 10.1038/bjc.2015.163 PMC4580380 26010411

[187] Huang Y Benaich N Tape C Kwok HF Murphy G. Targeting the Sheddase Activity of ADAM17 by an Anti-ADAM17 Antibody D1(A12) Inhibits Head and Neck Squamous Cell Carcinoma Cell Proliferation and Motility Via Blockage of Bradykinin Induced HERs Transactivation. Int J Biol Sci (2014) 10:702–14. 10.7150/ijbs.9326 PMC4081605 25013379

[188] Peng L Cook K Xu L Cheng L Damschroder M Gao C. Molecular Basis for the Mechanism of Action of an anti-TACE Antibody. mAbs (2016) 8:1598–605. 10.1080/19420862.2016.1226716 PMC5098442 27610476

[189] Mishra HK Pore N Michelotti EF Walcheck B. Anti-ADAM17 Monoclonal Antibody MEDI3622 Increases IFNγ Production by Human NK Cells in the Presence of Antibody-Bound Tumor Cells. Cancer Immunol Immunother (2018) 67:1407–16. 10.1007/s00262-018-2193-1 PMC6126979 29978334

